# One-year ecological momentary assessment of alcohol use, mood, and stress among individuals with alcohol use disorder during SARS-CoV-2 pandemics: a gender-specific reflection

**DOI:** 10.1007/s00406-024-01930-9

**Published:** 2024-11-19

**Authors:** Julia G. Wenzel, Markus Reichert, Hilmar Zech, Friederike Wedemeyer, Friederike Deeken, Gianna Spitta, Patrick Bach, Bernd Lenz, Ulrich W. Ebner-Priemer, Falk Kiefer, Michael A. Rapp, Henrik Walter, Andreas Heinz, Tobias Banaschewski

**Affiliations:** 1 Department of Psychiatry and Neurosciences, Charité-Universitätsmedizin Berlin, corporate member of Freie Universität Berlin and Humboldt-Universität Zu Berlin, Berlin, Germany; 2 Department of Psychiatry and Psychotherapy, Medical Faculty Mannheim, Central Institute of Mental Health, University of Heidelberg, Mannheim, Baden-Wuerttemberg Germany; 3 Mental mHealth Lab, Department of Sports and Sports Science, Karlsruhe Institute of Technology (KIT), Karlsruhe, Baden-Wuerttemberg Germany; 4 Department of eHealth and Sports Analytics, Faculty of Sports Science, Ruhr University Bochum (RUB), Bochum, North Rhine-Westphalia Germany; 5 Department of Psychiatry and Psychotherapy, Technische Universität Dresden, Dresden, Brandenburg Germany; 6 Department of Child and Adolescent Psychiatry, Psychosomatics and Psychotherapy, Centre of Mental Health, University of Würzburg, Bavaria, Germany; 7 Department of Psychiatry, Psychiatric University Hospital Charité at St. Hedwig Hospital, Charité-Universitätsmedizin Berlin, Berlin, Germany; 8 Department of Social and Preventive Medicine, University of Potsdam, Potsdam, Brandenburg Germany; 9 Department of Addictive Behavior and Addiction Medicine, Medical Faculty Mannheim, Central Institute of Mental Health (CIMH), University of Heidelberg, Mannheim, Baden-Wuerttemberg Germany; 10 mHealth Methods in Psychiatry, Department of Psychiatry and Psychotherapy, Medical Faculty Mannheim, Central Institute of Mental Health, Heidelberg University, Heidelberg, Baden-Wuerttemberg Germany; 11 Department of Clinical Psychology, Medical Faculty Mannheim, Central Institute of Mental Health (CIMH), University of Heidelberg, 68159 Mannheim, Baden-Wuerttemberg Germany

**Keywords:** Alcohol consumption, Mood, Stress, Ecological momentary assessment, Gender, Lockdown

## Abstract

Alcohol consumption (AC) is a leading risk factor for death, morbidity, and disability worldwide. Gender-specific differences in AC and its moderators, which may serve as markers for preventing severe alcohol use disorders (AUD), showed inconsistent results. Additionally, the impact of COVID-19-related lockdowns on these differences remains unclear. We examined gender-specific differences in short- and long-term factors affecting AC in individuals at risk for alcohol dependence, focusing on mood, stress, and the influence of restriction-dependent lockdown phases. 358 subjects with AUD aged 16 to 65 were studied over one year. Daily electronic diaries and monthly questionnaires were conducted from 10/01/2020 to 09/30/2021, assessing real-world trajectories of AC, mood (MDMQ), and stress (PSS-10) during Germany’s second COVID-19 wave. Multi-level models were used to assess associations between these measures and with several within- and between-subject variables. During lockdown, women experienced lower and even decreasing mood (valence: β = − 0.2, *p* < .039; calmness: β = − 0.3, *p* < .010), while men’s mood increased from the most restrictive lockdown phase (valence: β = 0.2, *p* < .001; calmness: β = 0.3, *p* < .001) to post-lockdown (valence: β = 0.5, *p* < .001; calmness: β = 0.6, *p* < .001). Stress increased earlier (β = 0.8, *p* < .001) and more prolonged (β = 0.4, *p* = .021) in women than in men. For both genders, daily mood was positively associated with daily AC (valence: β = 0.6, *p* = .004; calmness: β = 0.4, *p* = .013), leading to stronger drinking on days with elevated mood. Conversely, average mood was negatively associated with average AC (valence: β = − 1.6, *p* = .011; calmness: β = − 1.2, *p* = .041), indicating higher overall consumption with worse overall mood. Our findings highlight the need for interventions targeting mental distress in women with AUD during pandemics, as this group faces increased mental burden during social isolation and increased risk of alcohol dependence during persistent distress.

**Supplementary Information:**

The online version contains supplementary material available at 10.1007/s00406-024-01930-9.

## Introduction

Alcohol consumption (AC) is prevalent worldwide and is among the leading risk factors for death, morbidity, and disability [1, 2]. Harmful AC accounts for approximately 5.3% of all deaths [2]. Sex and gender-related differences in AC have been found repeatedly. For example, women drink less per drinking occasion, are less often problematic drinkers, and less often engaged in heavy episodic drinking [2]. These differences cannot be fully explained by gender-typical differences in body weight and constitution [2]. Moreover, the influence of presumed moderators of AC was found to differ between women and men [3]. In this study, we focused on mood and stress as moderators of AC associated with gender-related differences and the influence of COVID-19-related lockdown phases.

Studies suggested a gender-specific influence of mood and stress on heavy drinking. In that, women tend to drink more heavily when experiencing and coping with negative emotions, psychological distress, stress, or following interpersonal conflicts [4–8], in particular if a problematic drinking behavior [9, 10] or depression [11] is underlying. Men drank more on drinking days with positive mood [12], general pleasant feelings, but also in response to social pressure [13–16].

During the SARS-CoV-2 pandemic and particularly during lockdown, increased psychological distress, anxiety, depression, stress and loneliness, resulting from perceived social isolation [17, 18], were repeatedly reported in general, but especially in women [19–26]. Here, as opposed to men, women showed a positive association between COVID-19-related psychological distress (including self-reported depressed mood) and drinking quantity [27].

However, inconsistent results on gender-specific influences of mood [4, 28–30] and stress on AC [14, 29] suggest more complex relationships and the influence of other factors, e.g. age, social factors, and drinking motivation. Additionally, the large variability of methods and samples as well as retrospective ratings and low temporal resolutions in mood, stress and AC assessments might have contributed to inconsistent results [31].

To examine associations between time-varying variables, state-specific recall-bias needs to be considered. Therefore, ecological momentary assessment (EMA) and daily e-diaries are preferable methods [32]. Previous EMA-studies found positive associations of positive mood with AC and negative associations of negative mood with AC [12, 33, 34]. Gender differences were only observed when response inhibition capacity was considered. Hence, contrary to men, women with high response inhibition showed diminished positive associations of positive mood with AC and women with low response inhibition showed positive associations of negative pre-drinking mood with AC [12].

We conducted a one-year study with participants with mostly moderate AUD using daily e-diaries and monthly questionnaires. The objective was to examine gender-specific differences in short- and long-term factors that interact with AC in a sample at risk for alcohol dependence focusing on mood and stress. We further assessed the influence of restriction-dependent lockdown phases (pre-lockdown, lockdown light1, lockdown hard, lockdown light2, post-lockdown) as additional stressors during the second wave of SARS-CoV-2 pandemic in Germany.

According to previous results, we assumed greater impairments in women than in men during lockdown. Hence, we hypothesized lower mood and increased stress in women and a moderation of these gender differences by restriction-dependent lockdown phases. We further hypothesized different association directionalities in women and men, with negative associations of mood with AC and positive associations of perceived stress with AC among women, and positive associations of mood with AC and negative associations of perceived stress with AC among men. Additionally, we assumed a moderation of these gender-specific differences by restriction-dependent lockdown phases.

## Methods

### Subjects

Subjects were recruited at three sites in Germany (Charité Universitätsmedizin Berlin, Technical University Dresden, and Central Institute of Mental Health in Mannheim) as part of the Collaborative Research Center grant 265 “ReCoDe” (Losing and regaining control over drug intake) [35] (eAppendix 1). Included were individuals aged 16–65 years who met 2–9 AUD criteria according to DSM-5 (*The Diagnostic and Statistical Manual of Mental Disorders*) [36] without experiencing withdrawal symptoms, medically supervised alcohol withdrawal, or desire for therapeutic intervention. Each potential subject underwent an extensive diagnostic interview using SCID-5 (structured clinical interview according to DSM-5) to ensure study eligibility. A diagnosis of bipolar I disorder, psychotic disorder, schizophrenia, schizophrenic spectrum disorder, or substance use disorders (SUD) not due to alcohol, nicotine, or cannabis led to study exclusion. Subjects provided written informed consent and were financially compensated for their participation.

### Data acquisition

Along with various other assessments (eAppendix 2), a one-year follow-up was conducted using e-diaries via a smartphone application (movisensXS app; movisens GmbH, Germany) and a monthly acquired *Perceived Stress Scale* (PSS-10) [37, 38].

Data acquisition started in February 20, 2020. To ensure a sufficiently large sample (eAppendix 3) and to investigate lockdown-related influences, the current analyses cover October 01, 2020 to September 30, 2021. After excluding subjects with a compliance < 10% and study participation time of less than two weeks, the final sample included 358 subjects.

### E-diary items

E-diaries were used to assess real-life AC and mood (eAppendix 4). The subjects were asked to complete the e-diary every second day.

AC was determined by the number of alcoholic drinks consumed on the previous two days using a list of drinks of varying sizes (eTable 1). Based on this, the amount of alcohol consumed was calculated in grams.

Mood was acquired using two valence and two calmness items. These were based on the German *Multidimensional Mood Questionnaire* (MDMQ) [39] and were developed and validated for EMA and e-diaries. The final mood measures were calculated as separate calmness and valence sum scores.

### COVID-19 lockdown definition

The observation period covered the second COVID-19-related lockdown in Germany, as well as pre- and post-phases and was divided into 5 sections based on the extent of government actions to mitigate the SARS-CoV-2 pandemic: pre-lockdown (October 1–November 1, 2020), lockdown light1 (November 2–December 15, 2020), lockdown hard (December 16, 2020–February 28, 2021), lockdown light2 (March 1–May 8, 2021), and post-lockdown (May 9–September 30, 2021 (eTable 2).

### Statistical analyses

Multi-level models were used to examine the hierarchical time series data of the outcome variables AC, mood (valence and calmness), and stress (PSS), using their repeated measurements (level 1) nested within each subject (level 2). Additional time-varying categorical variables (level 1) were added to the models as predictors, including restriction-dependent lockdown phases, weekends, and holidays. Mood and stress measures were also used as predictor variables for AC to investigate associations with AC. The subject-level covariates gender, age, fulfilled AUD criteria, depression diagnosis (former/current), profession, highest school qualification, marital status, having at least one child and study site were added to the models to control for them.

Beside these main models for AC, valence, calmness, and stress prediction (eAppendix 5) several smaller models, e.g. containing only one predictor (basic models) or moderation analyses, were used to investigate the influence of certain variables on the outcome variables in more detail (see Supplement). Analyses were performed using R-4.2.1 [40].

## Results

### Participants

The 358 included subjects were aged between 17 and 65 years (M: 37.5, SD = 12.6, IQR: 27–48), met 2–9 AUD criteria (M = 4.1, SD = 1.6, IQR: 3–5) and consisted of 126 women (35.2%) and 232 men (64.8%) (see Tab. 1 and eTable 3 for more details). Both genders did not differ significantly in age (t(356) = -0.6, p = .546), met AUD criteria (t(356) = 1.13, p = .261), and depression diagnosis (χ²(1, 357) = 3.64, p = .057), although the proportion of current/former depression diagnoses was considerably higher among women (31.7%) than men (22.4%) (Table 1).

**Table 1:** Participant characteristics across the sample and separated by gender

Participant characteristics	Sample (N = 358)	Females (N = 126)	Males (N = 232)
Age, median (IQR) [range]	35 (27–48) [17–65]	34 (26–48) [17–62]	36 (28–48) [17–65]
AUD criteria, median (IQR) [range]	4 (3–5) [2-9]	4 (3–5) [2-9]	4 (3–5) [2-9]
AUD criteria			
2	63 (17.6%)	17 (13.5%)	46 (19.8%)
3	86 (24.0%)	32 (25.4%)	54 (23.3%)
4	64 (17.9%)	18 (14.3%)	46 (19.8%)
5	66 (18.4%)	30 (23.8%)	36 (15.5%)
6	50 (14.0%)	19 (15.1%)	31 (13.4%)
7	23 (6.4%)	9 (7.1%)	14 (6.0%)
8	3 (0.8%)	0 (0.0%)	3 (1.3%)
9	3 (0.8%)	1 (0.8%)	2 (0.9%)
Depression (former or current)	92 (25.7%)	40 (31.7%)	52 (22.4%)
Current profession	282 (78.8%)	101 (80.2%)	181 (78.0%)
Highest school qualification			
No school degree	0 (0.0%)	0 (0.0%)	0 (0.0%)
Pupil at a general education school	11 (3.1%)	5 (4.0%)	6 (2.6%)
Currently enrolled in career-based training	1 (0.3%)	0 (0.0%)	1 (0.4%)
Secondary general school certificate	6 (1.7%)	3 (2.4%)	3 (1.3%)
General certificate of secondary education	60 (16.8%)	22 (17.5%)	38 (16.4%)
Polytechnic secondary school	5 (1.4%)	1 (0.8%)	4 (1.7%)
Advanced technical college certificate	33 (9.2%)	11 (8.7%)	22 (9.5%)
General certificate of education	225 (62.8%)	79 (62.7%)	146 (62.9%)
Other	4 (1.1%)	0 (0.0%)	4 (1.7%)
Marital status			
Single	167 (46.6%)	62 (49.2%)	105 (45.3%)
Living in marriage or partnership	144 (40.2%)	47 (37.3%)	97 (41.8%)
Living separately	14 (3.9%)	4 (3.2%)	10 (4.3%)
Divorced	17 (4.7%)	6 (4.8%)	11 (4.7%)
Widowed	3 (0.8%)	2 (1.6%)	1 (0.4%)
Having at least one child	117 (32.7%)	36 (28.6%)	81 (34.9%)

### Alcohol consumption

**Fig. 1:**
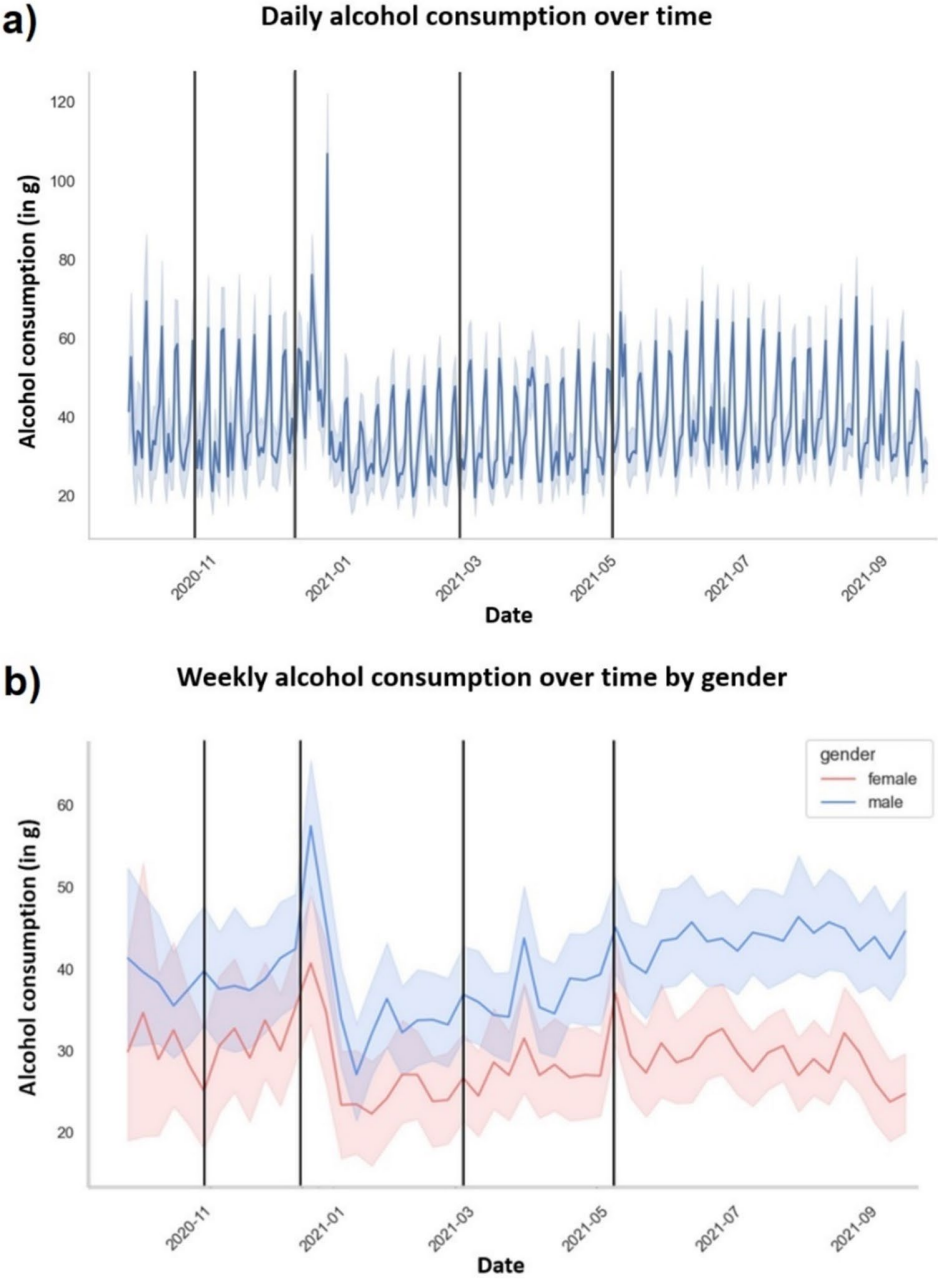
Real-life assessment of alcohol consumption. Showing fluctuations on **a** a daily level with a stable pattern of stronger consumption on weekends vs. weekdays and on **b** an aggregated weekly level for women (red) and men (blue) separately. Both plots included confidence intervals and vertical black lines representing the boundaries between different lockdown phases

Subjects consumed on average 37.2 g/d alcohol (95% CI [34.7, 39.7]) (~ 400 mL red wine). As illustrated in Fig. 1a), we found a stable pattern for AC on weekends compared to weekdays, which was characterized by an average increase in the amount of consumed alcohol by 14.7 g/d (95% CI [14.0, 15.4], *p* < 0.001) on weekends versus weekdays (eTable 4). On holidays we observed an increase of AC by 9.1 g/d (95% CI [7.6, 10.5], *p* < 0.001) compared to days without holiday. Both effects were significant in the main model (eTable 5).

Average daily AC in men was significantly higher (β = 13.10; 95% CI [8.0, 18.2], *p* < 0.001) than in women (see Fig. 1b), also in the main model (eTable 5). Women consumed on average 28.7 g/d (95% CI [24.6, 32.8]) (~ 300 mL red wine) and men 41.8 g/d (95% CI [38.4, 45.2]) (~ 430 mL red wine) (eTable 4). Significant weekend and holiday patterns were found for both genders (eTable 6, 7), but significantly stronger in men.

AC decreased by 2.3 g/d during lockdown light1 (95% CI [− 4.5, − 0.1], *p* = 0.044), by 5.2 g/d during lockdown hard (95% CI [− 7.2, − 3.1], *p* < 0.001), and by 5.7 g/d during lockdown light2 (95% CI [− 7.8, − 3.7], *p* < 0.001) compared to pre-lockdown (eTable 4), which was confirmed by the main model for lockdown hard and light2 (eTable 5).

### Mood

The average mood score was 10.4 for valence (95% CI [10.1, 10.6]) and 10.0 for calmness (95% CI [9.8, 10.2]). Similar to AC, we observed an increase on weekends (valence: β = 0.11, 95% CI [0.1, 0.1], *p* < 0.001; calmness: β = 0.18, 95% CI [0.1, 0.2], *p* < 0.001) and holidays (valence: β = 0.10, 95% CI [0.0, 0.2], *p* = 0.008; calmness: β = 0.20, 95% CI [0.1, 0.3], *p* < 0.001) (eTable 8, 9). All effects were significant in the main models (eTable 10, 11).

**Fig. 2:**
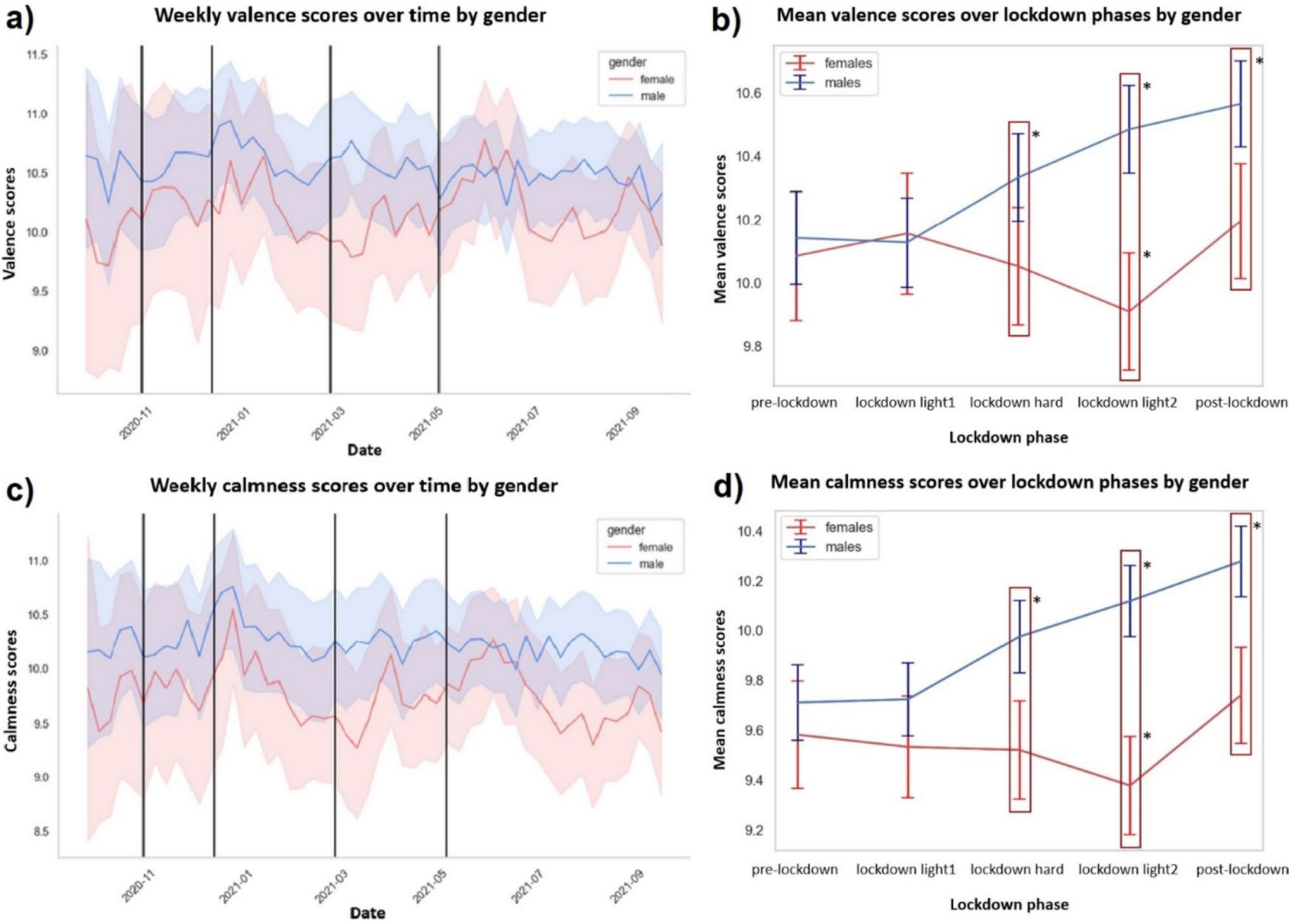
Real-life assessment of mood ratings (valence and calmness). Showing **a** valence and **c** calmness fluctuation (including confidence intervals) on an aggregated weekly level for women (red) and men (blue) separately as well as **b** valence and **d** calmness aggregated as mean values per lockdown phase (including standard errors) separated for women (red) and men (blue). Vertical black lines represent the boundaries between different lockdown phases. Red frames highlight significant differences between both genders, and black asterisks mark significant mood score changes per lockdown phase within each gender group relative to pre-lockdown (eTable 13)

Gender-specific fluctuations in valence and calmness over the one-year observation period are visualized in Fig. 2a and c. As illustrated in Fig. 2b and d, we found significant effects of lockdown phase on these mood measures (eTable 8, 9), which were moderated by gender (eTable 12).Thus, while women’s mood was lower and even decreased during lockdown light2 (valence: β = -0.20, 95% CI [-0.4, 0.0], p < .039; calmness: β = -0.27, 95% CI [-0.5, -0.1], p < .010), men’s mood increased continuously during lockdown hard (valence: β = 0.21, 95% CI [0.1, 0.3], p < .001; calmness: β = 0.27, 95% CI [0.2, 0.4], p < .001), lockdown light2 (valence: β = 0.37, 95% CI [0.3, 0.5], p < .001; calmness: β = 0.42, 95% CI [0.3, 0.6], p < .001), and post-lockdown (valence: β = 0.47, 95% CI [0.4, 0.6], p < .001; calmness: β = 0.59, 95% CI [0.5, 0.7], p < .001) (eTable 13).

Exploratory findings included a negative association of fulfilled AUD criteria with mood and lower mood scores with depression (eTables 10, 11).

### Perceived stress

**Fig. 3:**
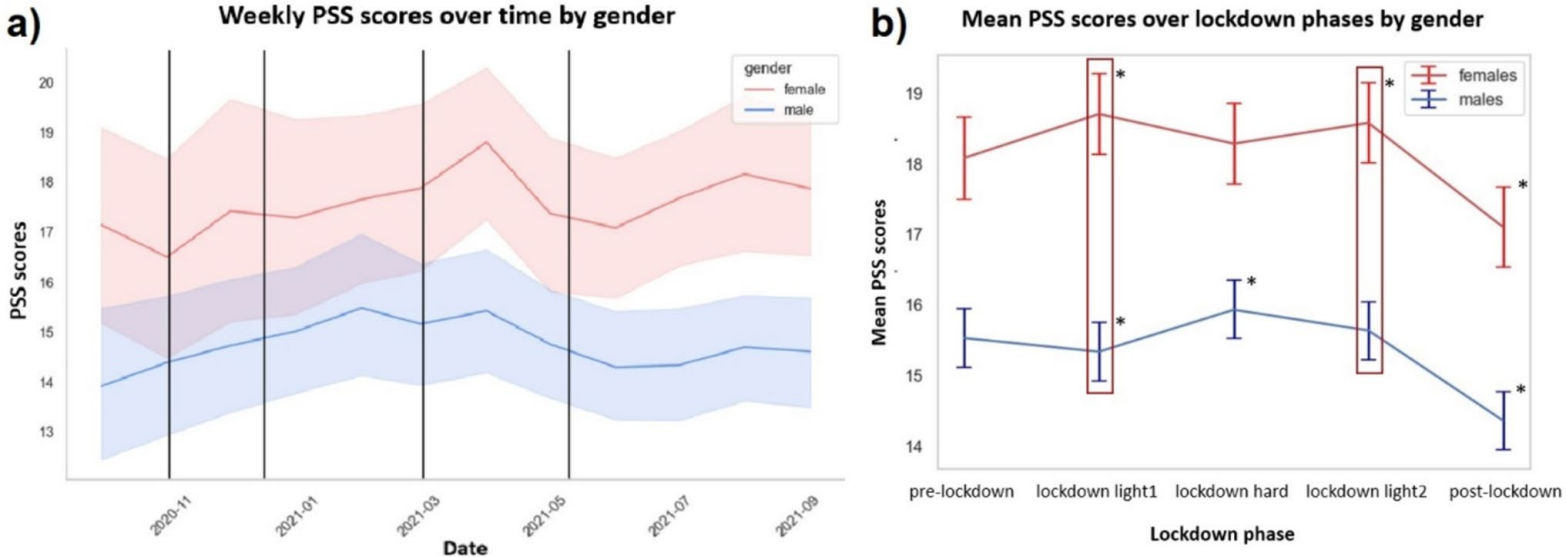
Real-life assessment of perceived stress. Showing fluctuation (including confidence intervals), **a** on an aggregated weekly level for women (red) and men (blue) separately and **b** aggregated as mean values per lockdown phase (including standard errors) separated for women (red) and men (blue). Vertical black lines represent the boundaries between different lockdown phases. Red frames highlight significant differences between both genders, and black asterisks mark significant PSS score changes per lockdown phase within each gender group relative to pre-lockdown (eTable 17)

The average PSS score was 15.9 (95% CI [15.2, 16.5]). Men showed significantly lower scores than women (β = − 2.78, 95% CI [− 4.1, − 1.4], *p* < 0.001) (eTable 14) (see Fig. 3a), which remained significant in the main model (eTable 15).

Both genders showed increasing PSS scores during lockdown and decreasing during post-lockdown (eTable 14). As illustrated in Fig. 3b), we found significant interactions between gender and lockdown phase (eTable 16). While women’s stress increased during lockdown light1 (β = 0.54, 95% CI [0.2, 0.9], *p* = 0.002) and light2 (β = 0.49, 95% CI [0.2, 0.8]; *p* = 0.002), men’s stress decreased during lockdown light1 (β = − 0.24, 95% CI [− 0.5, − 0.0]; *p* = 0.031) and did not change significantly during lockdown light2 (eTable 17).

Exploratory findings include a positive association of fulfilled AUD criteria with perceived stress and higher stress in subjects with depression (eTable 15).

### Associations of mood and stress with AC

Overall, we found positive associations of mood with AC (valence: β = 0.57, 95% CI [0.18, 0.96], *p* = 0.004; calmness: β = 0.42, 95% CI [0.1, 0.8], *p* = 0.013) (eTable 18, 19) with no moderation by gender or lockdown phase (eTable 20, 21).

Disentangling between- and within-subject associations of mood with AC revealed for both genders a negative between-subject association of individual average mood levels with individual average AC levels (valence: β = − 1.61, 95% CI [− 2.9, − 0.4], *p* = 0.011; calmness: β = − 1.24, 95% CI [− 2.4, − 0.1], *p* = 0.041) and a positive within-subject association of daily mood ratings and daily AC (valence: β = 0.57, 95% CI [0.2, 1.0], *p* = 0.004; calmness: β = 0.42, 95% CI [0.1, 0.8], *p* = 0.013) (eTable 22). Hence, individuals with high average mood drank on average less alcohol than individuals with low average mood (between-subject/cross-sectional level), while at days with increased mood individuals drank more compared to days with decreased mood (within-subject/intraindividual level). After splitting the sample using the average mood median, significant more AUD criteria (valence: t(178) = − 4.66, *p* < 0.001; calmness: t(178) = − 3.81, *p* < 0.001) were met in the low average mood subsample (valence: M = 4.5, SD = 1.5; calmness: M = 4.5, SD = 1.5) than in the high average mood subsample (valence: M = 3.8, SD = 1.6; calmness: M = 3.8, SD = 1.6).

In addition, no associations of perceived stress with AC (eTable 22, 23) and no moderation by gender or lockdown phase on potential associations (eTable 20, 21) were observed.

## Discussion

In the current one-year study, we used a high-frequency tracking approach to assess real-world trajectories of AC, mood, and perceived stress during the second SARS-CoV-2 wave in Germany in a sample at increased risk for alcohol dependence.

We found similar patterns as Deeken et al. (2022) [41], who examined data from a subsample of 189 participants collected from October 2, 2020, to February 28, 2021. In that, we observed substantially more AC on weekends and holidays. However, compared to women, men showed larger increases of AC on weekends and holidays. Since we also found increased mood scores on weekends and holidays, the larger AC increase in men is consistent with previous findings that men tend to drink more when they experience pleasant feelings and positive mood [12, 13]. However, this larger increase in men may also be driven by generally heavier AC during dinking occasions in men, consistent with WHO observations [2]. In contrast to other scientific and public debates regarding the effects of COVID-19 on health behaviors [42–44] and in line with Deeken et al. (2022) [41], we observed a decrease in AC during lockdown in both genders. Since this decrease began during the hard lockdown, which extended from mid-December 2019 to the end of February 2020, it seems reasonable to assume that this change in AC was primarily driven by seasonal factors related to New Year’s resolutions. However, this decrease disappeared once the lockdown ended in May 2020, suggesting an influence of lockdown-related restrictions on AC (e.g., fewer opportunities to drink caused by closed bars, clubs, and restaurants). Hence, even though the specific moderation of this AC decrease is unclear, it can be assumed that for some participants, resolutions might have been the intended starting point for a sustained reduction in AC, as reported by Deeken et al. (2022) [41], and that both genders of our at-risk sample had sufficient control over their AC despite the present lockdown-related stressors.

As hypothesized, we found lower mood and higher stress levels in women than in men and a moderation by restriction-dependent lockdown phases. This was characterized by an accumulative worsening of women’s mood during lockdown. In contrast, men showed a successive improvement of mood, which already occurred during the most restrictive lockdown phase. Consistent with previous findings, stress ratings were generally higher in women than in men [38, 45, 46]. Beyond that, we observed that women’s stress level increased earlier and to a greater extent over the course of the lockdown than for men. Nevertheless, men also experienced a strong increase of their stress level, although this was limited to the lockdown phase with the strongest restrictions (lockdown hard).

These findings suggest a greater and over time increasing burden on women with AUD during lockdown, while men’s mood and stress already improve during lockdown. This was in line with previous findings of lockdown-related increased negative mental health outcomes in women without AUD [19–22]. This greater impairment among women might be related to their increased responsibilities during lockdown, spending significantly more time on housework, childcare, and other unpaid work beside their own job, while their paid work decreased disproportionately compared to men, as did their work productivity when working from home [47]. Particularly the increase in housework and childcare led to greater mental distress already during the first lockdown compared to men [48]. Since 80% of our female sample were employed and almost 30% had at least one child (Tab. 1), a larger portion probably had to bear such increased responsibilities. Additionally, the pandemic-related increase in loneliness, for which women were among the most vulnerable [20], was associated with impaired mental health in general [49–51] and in women during pandemic in particular [52], which may have contributed to increased mental distress among women. Finally, more frequent gender-based violence during lockdown may also have affected women’s mental health [53–55].

Contrary to our second hypothesis and previous studies [4, 6, 27], we found no gender differences in associations of AC with mood or perceived stress, and no moderating influence of lockdown phase. Instead, we observed in both genders a drinking pattern that was frequently found in men, with heavier momentary AC on days with elevated mood [12–16]. Moreover, we observed a different directionality regarding the association between individual mean AC and individual mean mood. Specifically, we found that the lower the individual mean mood, the higher the individual mean AC. These differences between within- and between-level associations indicate an inherent complexity, which should be examined in more detail in future studies.

In contrast to earlier studies [9, 10], we found that women of our at-risk sample tended to consume more alcohol on days with elevated mood, just like men, even though these moments of high mood were significantly reduced during lockdown. Conversely, the lockdown-related increase in mental burden among the women did not lead to temporarily heavier AC. The increased responsibility of women during the lockdown [47] could, again, provide an explanation of our results. Hence, to maintain the required level of functionality, it may have been a conscious decision by the women not to respond to the increased burden with increased AC at the expense of their capacity. In fact, Deeken et al. (2022) [41] demonstrated a close coupling between drinking intention and AC in AUD individuals, suggesting that conscious abstention may have been used to maintain individual functioning.

It is assumed that the probability of experiencing a pandemic over the course of a lifetime (currently ~ 38%) will double in the coming decades, though it is currently unclear which pathogen will be the cause [56]. Given that pandemics are accompanied by various stressors (e.g. social Isolation, job loss, working hour reduction, financial constraints, childcare, home schooling, lacks of emotional and social support, loss of loved ones, impaired mental health) that often affect women in particular [20, 42, 47–52] and the important role of negative emotional states and stress in alcohol dependence among women [7, 8], it may be of strong interest to address mental distress of women with AUD during future pandemics through individual prevention, to avoid persistent mental burden and the development of dependence in this at-risk group.

### Strengths

We examined AC, mood and their associations in a rare and large sample of 358 subjects with mostly moderate AUD. This population is most at risk of escalating into severe alcoholism, but also might have the best chance of moving toward healthier drinking patterns. The high-frequency tracking approach allowed us to acquire a substantial amount of representative daily-life data points per subject over a period of up to one year. This resulted in a database that is exceptionally large and rare in comparison to other EMA and e-diary studies in this field. Numerous multi-level models were computed to investigate inherent relationships, potential moderators, and to control for confounding variables. Hence, the present study provides a unique and well-controlled in-depth insight into dynamics of AC and mood during the second wave of SARS-CoV-2 pandemic.

### Limitations

The current study has limitations that need to be considered. First, the retrospective assessment of mood and AC prevents clarifying whether a particular mood change led to a change in AC or vice versa. In that, high-resolution temporal dynamics cannot be disentangled using the present data.

Second, PSS scores were collected at a lower temporal resolution than the e-diary data (monthly) resulting in fewer and less representative stress measurements of real-life conditions. Although this significantly impaired the investigation of within-subject associations between stress and AC, global stress changes could still be investigated.

Third, since the observation period covers the second SARS-CoV-2 wave, our pre-lockdown phase was presumably affected by the first wave and might be considered as its post-lockdown phase. This may have influenced the assessment of lockdown phase effects, as our intercept (pre-lockdown) may have been biased. A recent study stated increased AC in 17% of subjects after the first wave and associations between increased AC and poorer general mental health, depressive symptoms and reduced psychological well-being. Although AC measures during pre-lockdown did not differ from post-lockdown, PSS scores during pre-lockdown were higher than during post-lockdown and men’s mood during post-lockdown was higher than during pre-lockdown, indicating a certain influence of the first wave.

Fourth, since the most restrictive lockdown phase (lockdown hard) included Christmas, New Year’s Eve and January (during which many people reduce their AC due to good New Year’s resolutions) a seasonal influence on the current results cannot be ruled out.

## Conclusion

In the current one-year study, we examined AC, mood and perceived stress, as well as the influence of gender and lockdown phase during the second SARS-CoV-2 wave in Germany in subjects with mostly moderate AUD using daily e-diaries and monthly questionnaires. We observed a stable pattern of significantly greater AC and enhanced mood on weekends and holidays, particularly evident in men. During lockdown, we found reduced AC and increased perceived stress in both genders as well as greater mental distress among women due to worse mood and a more pronounced stress increase. For both genders, momentary mood was positively associated with momentary AC, resulting in heavier drinking on days of elevated mood, while average mood was negatively associated with average AC, resulting in increased overall drinking the worse the general mood was. Indicating different directionalities of associations of short- and long-term mood factors with AC in both genders. These findings highlight the importance of addressing mood impairments during social isolation among the female AUD population through individualized prevention, as this group is more often exposed to greater mental burden during such times and may have an increased risk of developing alcohol dependence, especially if distress persists.

## Supplementary Information

Below is the link to the electronic supplementary material.

Supplementary file1 (PDF 1280 KB)
