# Supplementary material for: One-year ecological momentary assessment of alcohol use, mood, and stress among individuals with alcohol use disorder during SARS-CoV-2 pandemics: a gender-specific reflection
Source: Eur Arch Psychiatry Clin Neurosci. 2024 Nov 19;275(2):451–61. doi: 10.1007/s00406-024-01930-9 (PMC11910400; doi:10.1007/s00406-024-01930-9)
Supplement: Supplementary file 1 — Supplementary file1 (PDF 1280 KB) [file 406_2024_1930_MOESM1_ESM.pdf]

Supplementary Online Content

Article Title:

One-Year Ecological Momentary Assessment of Alcohol Use, Mood, and Stress among Individuals with Alcohol Use Disorder during SARS-Cov-2 Pandemic - a Gender-Specific Reflection

Journal Name:

European Archives of Psychiatry and Clinical Neuroscience

Author Names:

Julia G. Wenzel, DiplPsych[1#]; Markus Reichert, PhD[2,3,4]; Hilmar Zech, M.Sc.[5,6]; Friederike Wedemeyer, MD[7]; Friederike Deeken, PhD[8]; Gianna Spitta, MD[1]; Patrick Bach, MD[9]; Bernd Lenz, MD[9]; Ulrich W. Ebner-Priemer, PhD[3,10]; Falk Kiefer, MD[9]; Michael A. Rapp, MD, PhD[8]; Henrik Walter, MD, PhD[1]; Andreas Heinz, MD, PhD[1]; Tobias Banaschewski, MD, PhD[11#]

Affiliation:

[1] Department of Psychiatry and Neurosciences, Charité-Universitätsmedizin Berlin, corporate member of Freie Universität Berlin and Humboldt-Universität zu Berlin, Berlin, Germany.
[2] Department of Psychiatry and Psychotherapy, Central Institute of Mental Health, University of Heidelberg, Medical Faculty Mannheim, Mannheim, Baden-Wuerttemberg, Germany.
[3] Mental mHealth Lab, Department of Sports and Sports Science, Karlsruhe Institute of Technology (KIT), Karlsruhe, Baden-Wuerttemberg, Germany.
[4] Department of eHealth and Sports Analytics, Faculty of Sports Science, Ruhr University Bochum (RUB), Bochum, North Rhine-Westphalia, Germany.
[5] Department of Psychiatry and Neuroimaging Centre, Technische Universität Dresden, Dresden, Brandenburg, Germany.
[6] Department of Child and Adolescent Psychiatry, Psychosomatics and Psychotherapy, Centre of Mental Health, University of Würzburg, Bavaria, Germany
[7] Psychiatric University Hospital Charité at St. Hedwig Hospital, Campus Charité Mitte, Charité-Universitätsmedizin Berlin; Department of Psychiatry.
[8] Department of Social and Preventive Medicine, University of Potsdam, Potsdam, Brandenburg, Germany.
[9] Department of Addictive Behavior and Addiction Medicine, Central Institute of Mental Health (CIMH), Medical Faculty Mannheim, University of Heidelberg, Mannheim, Baden-Wuerttemberg, Germany.
[10] mHealth Methods in Psychiatry, Department of Psychiatry and Psychotherapy, Medical Faculty Mannheim, Central Institute of Mental Health, Heidelberg University, Heidelberg, Baden-Wuerttemberg, Germany.
[11] Department of Clinical Psychology, Central Institute of Mental Health (CIMH), Medical Faculty Mannheim, University of Heidelberg, Mannheim, Baden-Wuerttemberg, Germany.

Corresponding Authors:

Julia G. Wenzel, DiplPsych

Email: julia.wenzel@charite.de

Tobias Banaschewski, MD, PhD

Email: tobias.banaschewski@zi-mannheim.de

Table of Contents

eAppendix ... 3

eAppendix 1 Collaborative Research Center 265 "ReCoDe" ... 3

eAppendix 2 Detailed study procedure ... 4

eAppendix 3 Sample sizes per lockdown phase ... 5

eAppendix 4 Acquisition and preprocessing of the e-diary and PSS items ... 6

eAppendix 5 Statistical analyses and equations of the main models ... 7

eAppendix 6 Intraclass correlation coefficients ... 9

eFigures ... 10

eFigure 1 Sampling size trajectories across the five lockdown phases**.** ... 10

eTables ... 11

eTable 1 List of selectable alcoholic drinks in the e-Diary survey ... 11

eTable 2 Lockdown measures of the second wave of the SARS-CoV-2 pandemic in Germany ... 12

eTable 3 Participant and EMA characteristics across the sample and separated by gender ... 14

eTable 4 Results of basic multilevel models including only one variable for AC prediction ... 15

eTable 5 Results of the main multilevel model for AC prediction ... 17

eTable 6 Results of multilevel models examining the influence of weekends/holidays on AC and its moderation by gender ... 19

eTable 7 Results of moderation analyses gender * weekends/holidays for the outcome AC ... 21

eTable 8 Results of basic multilevel models including only one variable for valence prediction ... 22

eTable 9 Results of basic multilevel models including only one variable for calmness prediction ... 24

eTable 10 Results of the main multilevel model for valence prediction ... 26

eTable 11 Results of the main multilevel model for calmness prediction ... 28

eTable 12 Results of moderation analyses gender * lockdown phase for the outcome mood ... 30

eTable 13 Results of multilevel models examining the gender-specific influence of lockdown phases on valence and calmness ... 31

eTable 14 Results of basic multilevel models including only one variable for PSS prediction ... 32

eTable 15 Results of the main multilevel model for perceived stress as the outcome variable ... 34

eTable 16 Results of moderation analyses gender * lockdown phase for the outcome PSS ... 36

eTable 17 Results of multilevel models examining the gender-specific influence of lockdown phases on PSS 37

eTable 18 Results of multilevel models for the AC prediction by valence scores ... 38

eTable 19 Results of multilevel models for the AC prediction by calmness scores ... 39

eTable 20 Results of moderation analyses focusing on the influence of gender in AC prediction ... 40

eTable 21 Results of moderation analyses focusing on the influence of lockdown phase in AC prediction ... 41

eTable 22 Results of disentangling within- and between-person associations for AC prediction ... 43

eTable 23 Results of multilevel models for the AC prediction by PSS scores ... 44

eReferences ... 45

eAppendix

eAppendix 1 Collaborative Research Center 265 "ReCoDe"

The data for this study were collected as part of the Collaborative Research Center grant 265 "ReCoDe" (Losing and regaining control over drug intake). This was initiated in July 2019 across the three participating centers in Germany (Charité Universitätsmedizin Berlin, Technical University Dresden, and Central Institute of Mental Health in Mannheim).[1]

Three main objectives have been outlined for this research consortium: (1) to identify triggers and modifying factors that longitudinally modulate the trajectories of loss and regain of control over drug use in real life, (2) to investigate underlying behavioral, cognitive, and neurobiological mechanisms, and (3) to provide mechanism-based interventions. For the present study, data from before, during, and after the second wave of the SARS-CoV-2 pandemic in Germany, from October 01, 2020, to September 30, 2021, were used for analyses.

eAppendix 2 Detailed study procedure

Data collection was conducted at three sites in Germany (Charité - Universitätsmedizin Berlin, Technical University Dresden, and Central Institute of Health Mannheim) as part of a large, longitudinal cohort study within a research consortium focusing on substance use disorder (SUD)[1]. After an initial telephone screening, potentially eligible subjects participated at a baseline assessment that included an extensive diagnostic interview using SCID-5 (structured clinical interview according to DSM-5) for determining final study inclusion or exclusion. In addition, breath alcohol and drug tests were conducted. After inclusion, various baseline measurements e.g. socio-demographics, state and trait self-assessment questionnaires, MRI data, blood markers, and neuropsychological assessments and magnet resonance imaging (MRI) were performed. In addition, the smartphone application ("movisens" app; movisens GmbH, Germany) for the one-year longitudinal Ecological Momentary Assessment (EMA) was installed and initialized. Within one year of study participation, several additional assessments (e.g., questionnaires biomarkers, neuropsychological assessments) were conducted every fourth month as well as a COVID-19 focused questionnaires every month.

The study procedure was approved by the review boards of local ethics committee at the Charité – Universitätsmedizin Berlin (EA1/212/18), Technical University Dresden (EK 459112018), and Heidelberg University (2018-621N-MA). This study was realized accordingly to the Strengthening the Reporting of Observational Studies in Epidemiology (STOBE) reporting guidelines.

eAppendix 3 Sample sizes per lockdown phase

The recruitment for this study was continuous, so that the number of active participants, who were monitored over one year using ambulatory assessment, increased continuously over the course of study time.

Although data collection started in February 20, 2020, a sample size that provided sufficient statistical power was not reached until October 2020. This assumption can be inferred from the results of recent simulation studies[2] along with multilevel analysis guidelines[3]. Consequently, data collected prior to October 2020 could not be included in the pre-lockdown stage. Thus, to also investigate lockdown-related influences within a one-year period, the current analyses cover the period from October 01, 2020 to September 30, 2021. The initial sample in this assessment period comprised 400 subjects. After excluding subjects with less than 10% compliance and study participation time of less than two weeks, the final sample included 358 subjects.

Overall, the sample size in the pre-lockdown ranged from 85 to 112 subjects. In contrast, the sample size in Lockdown light 1 ranged from 112 to 140, in Lockdown hard from 140 to 185, in Lockdown light 2 from 185 to 226, and in Post-Lockdown from 226 to 291 subjects. The sampling size trajectories across the five lockdown phases were illustrated in eFigure 1.

However, according to current findings, multilevel models are generally well suited to deal with data structures that have varying amounts of data points.[3]

eAppendix 4 Acquisition and preprocessing of the e-diary and PSS items

E-diaries of the movisensXS app were used to assess real-life AC and mood e-diaries. Subjects installed the app on their smartphone or were provided with a study smartphone. During the one-year study, subjects were asked to complete the e-diary every second day by responding to the audible, vibrating, and visual signal that was triggered at 12 pm. This alarm could be moved up to 8 hours to 8 pm (at intervals of 5 minutes to 8 hours). In addition to these two-day queries, additional items on other topics were asked every 8 and 25 days. All items were presented in German language.

Alcohol consumption was recorded via the alcoholic drinks consumed on the previous two days individually. The items were formulated as follows: “Think about yesterday (following item: Think about the day before yesterday): Which and how many alcoholic drinks did you consume?” [„Denken Sie an gestern (following item: Denken Sie an vorgestern): Welche und wie viele alkoholische Getränke haben Sie konsumiert?“]. The subjects rated on a list of different alcoholic drinks with varying sizes (eTable 1) what drink they had consumed and the number of each. During preprocessing, these data were used to calculate the total amount of alcohol consumed (in grams) separately for each day. This resulted in a continuous data set with a daily resolution.

Mood was collected every other day. Here, at 12 noon, the mood within the past 24 hours was recorded as follows: "During the past 24 hours, I felt on average... satisfied - dissatisfied (valence: Item 1), restless - calm (calmness Item 1), uncomfortable - comfortable (valence Item 2), relaxed - tense (calmness Item 2)" [“In den letzten 24 Stunden fühlte ich mich im Durchschnitt… zufrieden – unzufrieden, unruhig – ruhig, unwohl – wohl, entspannt – angespannt”]. For each item, subjects rated on a bipolar 7-point Likert scale where their mood was between the two given descriptions. The final mood scores were calculated as sum score of both calmness items and both valence items (range: 2-14). Both scores were assigned to the current day as well as to the previous day, resulting in a continuous data set with a daily resolution.

Perceived stress was recorded by using the PSS-10 once a month for the previous four weeks. Subjects were asked to rate how regularly they experienced each of ten stressful situations on a 5-point scale (0 = “never”, 1 = ”almost never”, 2 = ”sometimes”, 3 = ”fairly often”, 4 = ”very often”). During preprocessing, we duplicated the acquired value for each day of the previous four weeks.

For all these outcome variables, both the individual mean-centered value (suffix _c) and the individual mean value for each subject (suffix _m) were calculated allowing within- and between-subject comparisons.

eAppendix 5 Statistical analyses and equations of the main models

Multi-level models were used to examine the hierarchical time series data of the outcome variables AC, mood, and perceived stress, using their repeated measurements (level 1) nested within each subject (level 2). The alpha level was set to 0.05 and the hypotheses were tested two-sided.

Additional time-varying categorical level 1 variables were added as predictors to the models, including restriction-dependent lockdown phases (0 = pre-lockdown, 1 = lockdown light 1, 2 = lockdown hard, 3 = lockdown light 2, 4 = post-lockdown), weekends (0 = Monday to Thursday, 1 = Friday to Sunday), and holidays (0 = no holiday on the current or following day, 1 = holiday on the current or following day).

It should be noted, that models using monthly acquired perceived stress as outcome did not include predictor variables with higher temporal resolution (weekend and holiday). Although mood (valence and calmness separately) and perceived stress served as outcome variables, these variables were also used as predictor variables for AC to investigate associations of mood with AC or perceived stress with AC. Consistent with established procedure[3], within-person mean centered versions of these variables were used as predictors. Moreover, subject-level covariates (level 2) such as gender, age, number of fulfilled AUD criteria, former or current depression diagnosis (0 = no, 1 = yes), current profession (0 = no, 1 = yes), highest school qualification, marital status, having at least one child (0 = no, 1 = yes) and study site (Berlin, Dresden, Mannheim) were added to the models.

Equations of the main models for the prediction of AC, valence, calmness, and perceived stress:

$$(1) Y(AC)_{ij} = \beta_{00} + \beta_{01} * age_j + \beta_{02} * gender_j + \beta_{03} * AUDcriteria_j + \beta_{04} * depression_j + \beta_{05} * profession_j + \beta_{06} * graduation_j + \beta_{07} * status_j + \beta_{08} * child_j + \beta_{09} * site_j + \beta_{10} * weekend_{ij} + \beta_{20} * holiday_{ij} + \beta_{30} * lockdown_{ij} + \beta_{40} * valence\_centered_{ij} + \beta_{40} * calmness\_centered_{ij} + \beta_{60} * perceivedstress\_centered_{ij} + \mu_{ij} + r_{ij}$$

$$(2) Y(valence)_{ij} = \beta_{00} + \beta_{01} * age_j + \beta_{02} * gender_j + \beta_{03} * AUDcriteria_j + \beta_{04} * depression_j + \beta_{05} * profession_j + \beta_{06} * graduation_j + \beta_{07} * status_j + \beta_{08} * child_j + \beta_{09} * site_j + \beta_{10} * weekend_{ij} + \beta_{20} * holiday_{ij} + \beta_{30} * lockdown_{ij} + \mu_{ij} + r_{ij}$$

$$(3) Y(calmness)_{ij} = \beta_{00} + \beta_{01} * age_j + \beta_{02} * gender_j + \beta_{03} * AUDcriteria_j + \beta_{04} * depression_j + \beta_{05} * profession_j + \beta_{06} * graduation_j + \beta_{07} * status_j + \beta_{08} * child_j + \beta_{09} * site_j + \beta_{10} * weekend_{ij} + \beta_{20} * holiday_{ij} + \beta_{30} * lockdown_{ij} + \mu_{ij} + r_{ij}$$

$$(4) Y(perceived\ stress)_{ij} = \beta_{00} + \beta_{01} * age_j + \beta_{02} * gender_j + \beta_{03} * AUDcriteria_j + \beta_{04} * depression_j + \beta_{05} * profession_j + \beta_{06} * graduation_j + \beta_{07} * status_j + \beta_{08} * child_j + \beta_{09} * site_j + \beta_{10} * lockdown_{ij} + \mu_{ij} + r_{ij}$$

Time-varying within-subject variables (level 1) with the subscript ij denote the reported value of the jth subject at the ith assessment timepoint. The intercept as well as the effects or slopes of the level 1 variables weekend, holiday, lockdown, valence, calmness and perceived stress are described by the beta coefficients. While r_{ij} represents the within-person variability it is also referred to as residuals.

Between-subject effects were estimated on level 2 using subject-level variables. Thus, effects of age, gender, number of fulfilled AUD criteria, depression diagnosis, having a current profession, highest school qualification, marital status, having at least one child and site on the individual AC, mood (valence and calmness), and perceived stress were represented by the respective beta coefficient. Subject-specific variation of effects, which lead to deviations around the fixed effects, is also referred to as subject-level random effects, here represented by μ_{ij}.

eAppendix 6 Intraclass correlation coefficients

To examine how much of the total variance in our variables under study was due to between-subject differences, we estimated the intraclass correlation coefficients (ICC) using unconditional models.

For AC, we obtained an ICC of 0.26 indicating that, similar to Deeken et al. (2022)[4], 74% of the variance in amounts of alcohol consumed was due to within-subject fluctuations (level 1). In comparison, the ICC for females was 0.2, which was lower than that for males, which found to be 0.26. Considering the mood variables, we observed an ICC of 0.53 for valence as well as calmness suggesting that for both mood variables, 47% of the variance was due to within-subject fluctuations (level 1). In the female sample, we found a lower ICC of 0.48 compared to the male sample with an ICC of 0.57 for both mood variables. For perceived stress, an ICC of 0.77 was found for the whole sample, indicating that 23% of the variance in the data was due to within-subject fluctuations (level 1). This finding reflects the lower temporal resolution of this variable compared to the e-diary variables with the daily resolution. With regard to gender, an ICC of 0.73 was observed for females and an ICC of 0.78 for males.

eFigures

eFigure 1 Sampling size trajectories across the five lockdown phases.
Continuous recruitment in this study resulted in an increasing number of data points across the respective blocking phases, which was shown separated by vertical red lines for all five phases below.

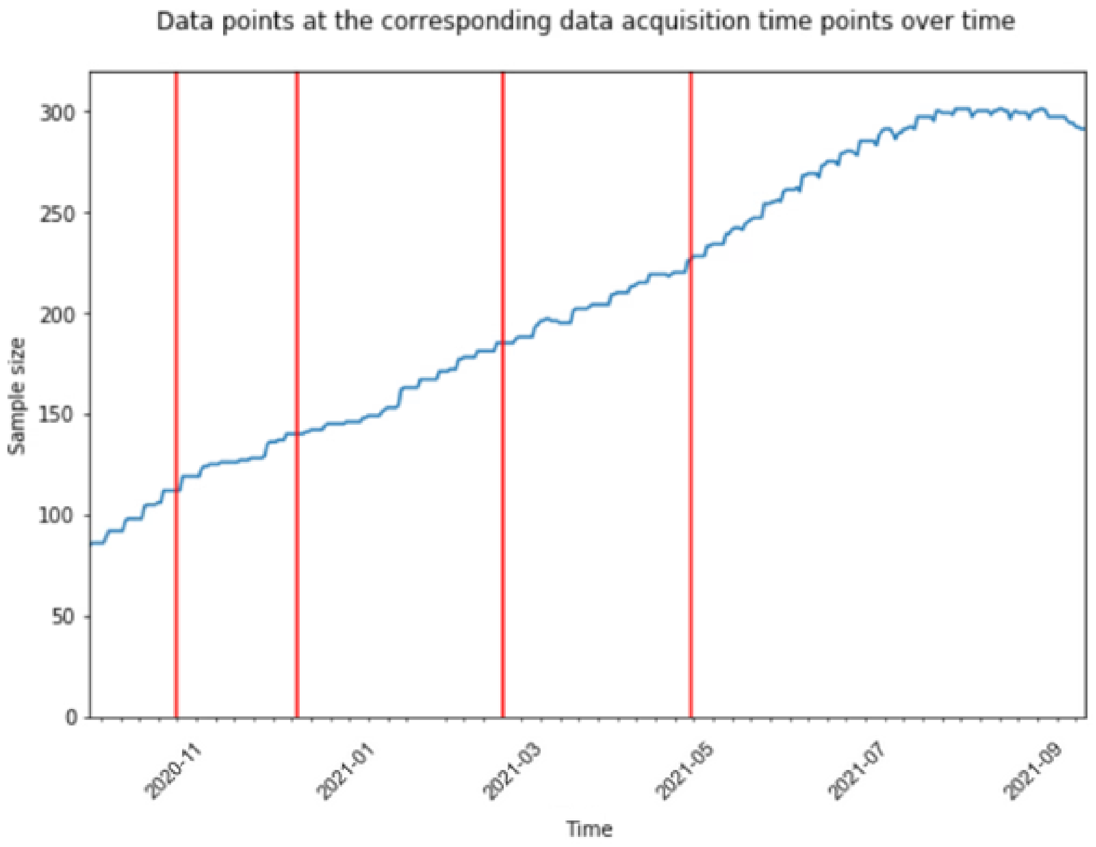

eTables

eTable 1 List of selectable alcoholic drinks in the e-Diary survey

Drink	Amount of Alcohol	Alcohol by Volume
No alcoholic drink	-	-
Small beer	0.2 l	5.0 %
Regular beer	0.33 l	5.0 %
Large beer	0.5 l	5.0 %
Small white wine	0.1 l	11.0 %
Regular white wine	0.2 l	11.0 %
Bottle of white wine	0.75 l	11.0 %
Small red wine	0.1 l	12.0 %
Regular red wine	0.2 l	12.0 %
Bottle of red wine	0.75 l	12.0 %
Sparkling wine	0.1 l	11.5 %
Bottle of sparkling wine	0.75 l	11.5 %
Fortified wine (e.g. port, Sherry)	5 cl	19.7 %
Small liqueur (e.g. Cointreau, Sambuca, Underberg, Jägermeister)	2 cl	20.0 %
Large liqueur (e.g. Cointreau, Sambuca, Underberg, Jägermeister)	5 cl	20.0 %
Sweet liqueur (e.g. Aperol, Amaretto, Advocaat, Baileys, Batida de Coco)	2 cl	17.0 %
Small spirit (e.g. fruit liqueur, cherry liqueur, Vodka, Gin, juniper, corn brandy)	2 cl	40.0 %
Large spirit (e.g. fruit liqueur, kirsch, Vodka, Gin, juniper, corn brandy)	5 cl	40.0 %
Spirit (e.g. fruit liqueur, kirsch, Vodka, Gin, juniper, corn brandy)	0.1 l	40.0 %
Small spirit (strong)	2 cl	65.0 %
Brandy (e.g. Brandy, Cognac, Armagnac, Metaxa)	2 cl	36.0 %

eTable 2 Lockdown measures of the second wave of the SARS-CoV-2 pandemic in Germany

Lockdown Phase	Lockdown Measures
**Pre-Lockdown** *(2020-10-01 to 2020-11-01)*	Following the AHA guideline (“**A**bstand, **H**ygiene und **A**lltagsmaske”; translated: distance, hygiene, and mask) was recommended.
**Lockdown light 1** *(2020-11-02 to 2020-12-15)*	In general, spending time in public was restricted to members of one's own and one other household. But the maximum number of people allowed to meet was limited to 10.<br>Institutions and facilities assigned to the recreational sector were closed, including recreational and amateur sport activities at and in public and private sports facilities of all kinds. Exceptions were individual sports alone, in pairs, or with members of the own household only.<br>In general, individuals were encouraged to refrain from non-essential private travel and visits, including to visiting relatives. Temporary accommodations for overnight trips within the country were provided only for necessary travel and strictly non-tourist purposes.<br>Restaurants, bars, clubs, pubs and similar places were closed. Exceptions include the delivery and collection of takeaway food intended for consumption at home as well as the operation of canteens at workplaces.[5]
**Lockdown hard** *(2020-12-16 to 2021-02-28)*	Private get-togethers were limited to a maximum of 5 people coming from a maximum of two households.<br>Retail and service businesses such as hairdressers and beauty salons were required to remain closed as of December 16. Exceptions were made for grocery stores, drug stores, pharmacies, opticians, gas stations, auto repair shops, banks, post offices, dry cleaners, and Christmas tree dealers, as well as medically necessary treatments (such as physical therapy).<br>At schools, contacts have been substantially restricted from December 16 to January 10, 2021. Children should be cared for at home during this time if possible. Therefore, schools generally remained closed or school attendance was suspended throughout this period. Emergency care was provided and remote learning was enabled. Similar arrangements were made for daycare centers. additional possibilities were provided for parents to take paid leave for child care.<br>Employers were requested to evaluate whether business sites could be closed either through company vacations or by providing generous home office solutions from Dec. 16 to Jan. 10, 2021.<br>In food services, delivery and pickup of take-out meals was still permitted. Consumption on site was not allowed. Drinking alcoholic drinks on public spaces was prohibited from Dec. 16 to Jan. 10. Violators were subject to fines.[6]
**Lockdown light 2** *(2021-03-01 to 2021-05-08)*	Since March 2021, initial relaxations of lockdown restrictions have taken place, the extent of which, however, was determined by each federal state depending on the respective infection situation. Accordingly, hair salons, nail salons, beauty salons and garden centers were allowed to reopen in certain regions.<br>Zoos, wildlife parks, botanical gardens, memorials, museums and galleries were reopened and certain sports activities were allowed (including indoor sports).<br>At the national level, more private contacts were permitted again. since March 8, bookstores, flower stores and garden markets were allowed to open. However, no opening steps were decided for the tourism and gastronomy sectors.<br>Retailers were able to reopen in certain regions under certain conditions (e.g., appointment shopping: "Click and Meet," mandatory masks).

	In the first half of March, domestic travel restrictions were lifted and on-site teaching was enabled.<br>At the end of March to the beginning of April, increased infection rates led to a temporary increase in lockdown restrictions in certain federal states, such as nighttime curfews. Negative Covid tests were required e.g. to enter certain stores or receive certain services.<br>Nationwide, the number of Corona vaccinations jumped. In early May, numerous easing measures for vaccinated people came into effect, so that a negative Covid test was no longer needed.<br>Travel to German vacation areas and visits to recreational parks were enabled.<br>In early May, the federal government allowed rapid easing of lockdown restrictions for fully vaccinated and recovered persons. As a result, contact and exit restrictions were reversed for these groups.[7-10]
**Post-Lockdown**<br>*(2021-05-09 to 2021-09-20)*	Easing of lockdown restrictions for vaccinated and recovered persons took effect: fully vaccinated persons or those recovered from Covid-19 now regained more freedom in Germany. Contact and exit restrictions were no longer imposed on them, and they were treated equally to people with negative Covid test results. Vaccinated and recovered people thus no longer needed negative tests to go shopping or to the hairdresser, for example.<br>The country's entry regulations were also revised to equalize those who had been tested and those who had been vaccinated. For example, quarantine obligations could be waived for fully vaccinated returning travelers.<br>Other easing measures also began to take effect. For example, gastronomy establishments were able to reopen gradually across the country.[7-10]

eTable 3 Participant and EMA characteristics across the sample and separated by gender

Participant and EMA Characteristics	Sample (N = 358)	Females (N = 126)	Males (N = 232)
**Age**, median (IQR) [range]	35 (27-48) [17-65]	34 (26-48) [17-62]	36 (28-48) [17-65]
**AUD criteria**, median (IQR) [range]	4 (3-5) [2-9]	4 (3-5) [2-9]	4 (3-5) [2-9]
**AUD criteria**			
2	63 (17.6%)	17 (13.5%)	46 (19.8%)
3	86 (24.0%)	32 (25.4%)	54 (23.3%)
4	64 (17.9%)	18 (14.3%)	46 (19.8%)
5	66 (18.4%)	30 (23.8%)	36 (15.5%)
6	50 (14.0%)	19 (15.1%)	31 (13.4%)
7	23 (6.4%)	9 (7.1%)	14 (6.0%)
8	3 (0.8%)	0 (0.0%)	3 (1.3%)
9	3 (0.8%)	1 (0.8%)	2 (0.9%)
**Depression** (former or current)	92 (25.7%)	40 (31.7%)	52 (22.4%)
**Current profession**	282 (78.8%)	101 (80.2%)	181 (78.0%)
**Highest school qualification**			
no school degree	0 (0.0%)	0 (0.0%)	0 (0.0%)
Pupil at a general education school	11 (3.1%)	5 (4.0%)	6 (2.6%)
Currently enrolled in career-based training	1 (0.3%)	0 (0.0%)	1 (0.4%)
Secondary general school certificate (Hauptschulabschluss)	6 (1.7%)	3 (2.4%)	3 (1.3%)
General Certificate of Secondary Education (Realschulabschluss)	60 (16.8%)	22 (17.5%)	38 (16.4%)
Polytechnic secondary school (Abschluss polytechnische Oberschule)	5 (1.4%)	1 (0.8%)	4 (1.7%)
Advanced technical college certificate (Fachhochschulreife)	33 (9.2%)	11 (8.7%)	22 (9.5%)
General Certificate of Education (Abitur)	225 (62.8%)	79 (62.7%)	146 (62.9%)
Another school degree	4 (1.1%)	0 (0.0%)	4 (1.7%)
**Marital status**			
Single	167 (46.6%)	62 (49.2%)	105 (45.3%)
Living in marriage or partnership	144 (40.2%)	47 (37.3%)	97 (41.8%)
Living separately	14 (3.9%)	4 (3.2%)	10 (4.3%)
Divorced	17 (4.7%)	6 (4.8%)	11 (4.7%)
Widowed	3 (0.8%)	2 (1.6%)	1 (0.4%)
**Having at least one child**	117 (32.7%)	36 (28.6%)	81 (34.9%)
**EMA compliance (in %)**			
AC (median (IQR) [range])	77.1 (52.9-93.1) [10.1-100]	78.9 (57.9-94.3) [10.6-100]	76.9 (51.8-92.7) [10.1-100]
Valence (median (IQR) [range])	75.9 (52.9-91.5) [8.8-99.6]	77.8 (56.4-92.6) [8.8-99.6]	75.8 (51.9-90.8) [10.1-99.4]
Calmness (median (IQR) [range])	75.9 (52.8-91.2) [8.8-99.6]	77.8 (56.4-92.6) [8.8-99.6]	75.8 (51.6-90.6) [10.1-99.4]

eTable 4 Results of basic multilevel models including only one variable for AC prediction Predictors comprised all within- (level 1) and between-subject (level 2) variables.

	**Basic models**<br>**Outcome: alcohol consumption**			
Predictor	β coefficient (SE) [95% CI]	t(df)	F-value	p-value
Mean AC	37.19 (1.28) [34.67 - 39.70]	28.98 (50709)	839.94	<.001
Gender				
Intercept (female)	28.72 (2.09) [24.63 - 32.81]	13.76 (50709)	897.20	<.001
Male	13.10 (2.60) [7.99 - 18.20]	5.04 (356)	25.44	<.001
Age				
Intercept (age = 17)	26.07 (3.98) [18.26 - 33.88]	6.54 (50709)	858.48	<.001
Age	0.30 (0.10) [0.10 - 0.49]	2.94 (356)	8.65	.004
AUD criteria				
Intercept (AUD = 2)	26.71 (3.50) [19.86 - 33.56]	7.64 (50709)	861.99	<.001
AUD criteria	2.54 (0.79) [0.99 - 4.09]	3.22 (356)	10.35	.001
Depression diagnosis				
Intercept (no depression)	36.37 (1.49) [33.45 - 39.29]	24.42 (50505)	834.59	<.001
Depression diagnosis	3.16 (2.96) [-2.65 - 8.98]	1.07 (355)	1.14	.286
Current profession				
Intercept (no job)	41.46 (3.06) [35.45 - 47.46]	13.53 (48844)	804.21	<.001
Current job	-5.20 (3.39) [-11.87 - 1.47]	-1.53 (343)	2.36	.126
Highest school qualification				
Intercept (no degree)	27.44 (7.39) [12.95 - 41.92]	3.71 (48844)	797.46	<.001
Pupil	1.50 (25.37) [-48.40 - 51.39]	0.06 (337)		.953
Current career-based training	-	-		-
Secondary general school certificate	13.49 (12.37) [-10.85 - 37.83]	1.09 (337)		.276
General Certificate of Secondary Education	9.70 (8.04) [-6.11 - 25.51]	1.21 (337)	0.80	.228
Polytechnic secondary school	23.51 (13.13) [-2.32 - 49.34]	1.79 (337)		.074
Advanced technical college certificate	4.45 (8.54) [-12.35 - 21.24]	0.52 (337)		.603
General Certificate of Education	10.56 (7.57) [-4.33 - 25.45]	1.40 (337)		.163
other school degree	15.73 (14.25) [-12.31 - 43.76]	1.10 (337)		.271
Marital status				
Intercept (single)	34.48 (1.87) [30.81 - 38.16]	18.40 (48844)	817.26	<.001
Marriage/partnership	4.61 (2.75) [-0.79 - 10.02]	1.68 (340)		.094
Living separately	0.70 (6.70) [-12.48 - 13.87]	0.10 (340)	2.58	.917
Divorced	8.88 (6.17) [-3.25 - 21.01]	1.44 (340)		.151
Widowed	36.88 (13.94) [9.46 - 64.31]	2.65 (340)		<.001
Having a child				
Intercept (no child)	34.46 (1.60) [31.32 - 37.59]	21.56 (48844)	819.36	<.001
At least one child	8.12 (2.75) [2.72 - 13.53]	2.96 (343)	8.75	.003
Study center				
Intercept (CI Mannheim)	34.84 (2.25) [30.44 - 39.24]	15.51 (50709)	841.25	<.001
Charité Berlin	5.83 (3.51) [-1.07 - 12.73]	1.66 (355)	1.38	.098
TU Dresden	2.29 (2.95) [-3.51 - 8.10]	0.78 (355)		.438
Weekend				

Predictor	Basic models<br>Outcome: alcohol consumption<br>β coefficient (SE) [95% CI]	t(df)	F-value	p-value
Intercept (weekday)	31.02 (1.29) [28.49 - 33.55]	24.04 (50708)	841.58	<.001
Weekend	14.72 (0.36) [14.02 - 15.41]	41.45 (50708)	1717.73	<.001
Holiday				
Intercept (no holiday)	36.75 (1.28) [34.23 - 39.27]	28.62 (50708)	839.09	<.001
Holiday	9.06 (0.75) [7.59 - 10.53]	12.05 (50708)	145.20	<.001
Lockdown phase				
Intercept (pre-lockdown)	39.82 (1.61) [36.66 - 42.97]	24.75 (50705)	842.01	<.001
Lockdown light 1	-2.28 (1.13) [-4.50 - -0.06]	-2.12 (50705)		.044
Lockdown hard	-5.17 (1.01) [-7.24 - -3.10]	-5.13 (50705)		<.001
Lockdown light 2	-5.74 (1.06) [-7.83 - -3.66]	-4.59 (50705)	25.13	<.001
Post-lockdown	-1.51 (1.05) [-3.56 - 0.54]	-0.61 (50705)		.148
Valence (centered)				
Intercept	36.63 (1.29) [34.11 - 39.15]	28.49 (46039)	810.15	<.001
Valence_c	0.57 (0.20) [0.18 - 0.96]	2.90 (46039)	8.40	.004
Calmness (centered)				
Intercept	36.64 (1.28) [34.12 - 39.15]	28.58 (46028)	812.45	<.001
Calmness_c	0.41 (0.17) [0.09 - 0.75]	2.47 (46028)	6.12	.013
Perceived Stress (centered)				
Intercept	37.04 (1.34) [34.41 - 39.67]	27.63 (35059)	764.87	<.001
PSS_c	-0.06 (0.12) [-0.30 - 0.17]	-0.55 (35059)	0.30	.585

Equation:

$$Y(AC)_{ij} = \beta_{00} + \beta_{01} * predictor_{(i)j} + \mu_{ij} + r_{ij}$$

eTable 5 Results of the main multilevel model for AC prediction
All within-subject (level 1) and between-subject (level 2) variables were included in one model.

	**Main model Outcome: alcohol consumption**			
Predictor	β coefficient (SE) [95% CI]	t(df)	F-value	p-value
intercept	4.54 (9.80) [-14.67 - 23.76]	0.46 (31128)	797.13	.642
Gender				
Female	0 [Reference]	0 [Reference]	17.80	0 [Reference]
Male	11.87 (2.78) [6.40 - 17.35]	4.27 (295)		<.001
Age	0.20 (0.15) [-0.11 - 0.50]	1.28 (295)	10.99	.203
AUD criteria	2.04 (0.86) [0.35 - 3.74]	2.37 (295)	8.11	.018
Depression diagnosis				
No depression	0 [Reference]	0 [Reference]	0.52	0 [Reference]
Depression diagnosis	2.04 (3.14) [-4.14 - 8.22]	0.65 (295)		.516
Current profession				
No current job	0 [Reference]	0 [Reference]	1.23	0 [Reference]
Current job	-4.27 (3.47) [-11.09 - 2.55]	-1.23 (295)		.219
Highest school qualification				
no school degree	0 [Reference]	0 [Reference]		0 [Reference]
Pupil	-1.22 (24.16) [-48.76 - 46.32]	-0.05 (295)		.960
Current career-based training	-	-		-
Secondary general school certificate	9.89 (12.38) [-14.48 - 34.26]	0.80 (295)		.425
General Certificate of Secondary Education	1.14 (8.76) [-16.11 - 18.39]	0.13 (295)	0.40	.897
Polytechnic secondary school	2.31 (13.38) [-24.02 - 28.64]	0.173 (295)		.897
Advanced technical college certificate	-1.86 (9.20) [-19.98 - 16.25]	-0.20 (295)		.840
General Certificate of Education	4.39 (8.22) [-11.79 - 20.58]	0.53 (295)		.594
other school degree	-1.69 (15.87) [-32.92 - 29.54]	-0.11 (295)		.915
Marital status				
Single	0 [Reference]	0 [Reference]		0 [Reference]
marriage/partnership	1.76 (3.58) [-5.28 - 8.80]	0.49 (295)		.623
Living separately	-6.53 (7.12) [-20.54 - 7.49]	0.49 (295)	2.43	.360
Divorced	1.22 (6.80) [-12.17 - 14.60]	0.18 (295)		.858
Widowed	37.33 (13.91) [9.96 - 64.69]	2.68 (295)		.008
Having a child				
No child	0 [Reference]	0 [Reference]	4.03	0 [Reference]
At least one child	6.44 (3.60) [-0.65 - 13.53]	1.79 (295)		.075
Study center				
CI Mannheim	0 [Reference]	0 [Reference]		0 [Reference]
Charité Berlin	4.33 (3.75) [-3.06 - 11.72]	1.15 (295)	0.87	.250
TU Dresden	0.93 (3.18) [-5.33 - 7.18]	0.29 (295)		.771
Weekend				
Weekday	0 [Reference]	0 [Reference]	989.34	0 [Reference]
Weekend	13.77 (0.44) [12.90 - 14.63]	31.22 (31128)		<.001
Holiday				

Predictor	Main model<br>Outcome: alcohol consumption<br>β coefficient (SE) [95% CI]	t(df)	F-value	p-value
no holiday	0 [Reference]	0 [Reference]	77.71	0 [Reference]
Holiday	9.33 (0.89) [7.58 - 11.08]	10.46 (31128)		<.001
Lockdown phase				
Pre-lockdown	0 [Reference]	0 [Reference]	31.38	0 [Reference]
Lockdown light 1	-0.21 (1.39) [-2.93 - 2.50]	-0.15 (31128)		.879
Lockdown hard	-6.75 (1.30) [-9.31 - -4.20]	-5.19 (31128)		<.001
Lockdown light 2	-5.91 (1.30) [-8.45 - -3.36]	-4.55 (31128)		<.001
Post-lockdown	-0.72 (1.29) [-3.25 - 1.81]	-0.56 (31128)		.577
Valence_c	0.77 (0.16) [0.45 - 1.09]	4.73 (31128)	19.27	<.001
Calmness_c	-0.33 (0.16) [-0.64 - -0.02]	-2.10 (31128)	4.89	.036
PSS_c	0.10 (0.07) [-0.03 - 0.23]	1.55 (31128)	2.41	.120

Equation:

$$Y(AC)_{ij} = \beta_{00} + \beta_{01} * age_j + \beta_{02} * gender_j + \beta_{03} * AUDcriteria_j + \beta_{04} * depression_j + \beta_{05} * profession_j + \beta_{06} * graduation_j + \beta_{07} * status_j + \beta_{08} * child_j + \beta_{09} * site_j + \beta_{10} * weekend_{ij} + \beta_{20} * holiday_{ij} + \beta_{30} * lockdown_{ij} + \beta_{40} * valence\_centered_{ij} + \beta_{40} * calmness\_centered_{ij} + \beta_{60} * perceivedstress\_centered_{ij} + \mu_{ij} + r_{ij}$$

eTable 6 Results of multilevel models examining the influence of weekends/holidays on AC and its moderation by gender

Both basic models with only one variable as predictor and larger models were used to control for the influence of between-subject variables age, AUD criteria, depression, and site.

	Gender-specific models of the influence of weekends and holidays<br>Outcome: alcohol consumption			
Predictor	β coefficient (SE) [95% CI]	t(df)	F-value	p-value
**Females (N = 126)**				
Basic multilevel models of the female sample using one variable (weekend or holiday) as predictor for AC				
Weekend				
Intercept (weekday)	24.25 (1.57) [21.16 - 27.33]	15.41 (18971)	339.37	<.001
Weekend	10.75 (0.49) [9.79 - 11.71]	21.88 (18971)	478.64	<.001
Holiday				
Intercept (no holiday)	28.44 (1.56) [25.38 - 31.05]	18.22 (18971)	339.37	<.001
Holiday	6.18 (1.04) [4.14 - 8.21]	5.94 (18971)	35.30	<.001
Multilevel model of the female sample using weekend/holiday as predictor for AC extended by between-person predictors and lockdown phase				
Intercept	8.78 (7.49) [-5.91 - 23.46]	1.17 (18966)	346.48	.241
Weekend				
Weekday	0 [Reference]	0 [Reference]	484.84	0 [Reference]
Weekend	11.21 (0.51) [10.21 - 12.21]	22.02 (17932)		<.001
Holiday				
no holiday	0 [Reference]	0 [Reference]	35.73	0 [Reference]
Holiday	6.49 (1.08) [4.36 - 8.61]	5.98 (17932)		<.001
**Males (N = 232)**				
Basic multilevel models of the male sample using one variable (weekend or holiday) as predictor for AC				
Weekend				
Intercept (weekday)	34.65 (1.73) [31.25 - 38.04]	20.00 (31736)	591.32	<.001
Weekend	17.09 (0.48) [16.13 - 18.04]	35.24 (31736)	1241.63	<.001
Holiday				
Intercept (no holiday)	41.30 (1.72) [37.92 - 44.68]	23.95 (31736)	588.66	<.001
Holiday	10.76 (1.03) [8.75 - 12.77]	10.48 (31736)	109.83	<.001
Multilevel model of the male sample using weekend/holiday as predictor for AC extended by between-person predictors and lockdown phase				
Intercept	2.46 (7.51) [-12.27 - 17.19]	0.33 (31527)	639.99	.743
Weekend				
Weekday	0 [Reference]	0 [Reference]	1243.72	0 [Reference]
Weekend	17.39 (0.49) [16.43 - 18.36]	35.26 (30706)		<.001
Holiday				
no holiday	0 [Reference]	0 [Reference]	111.77	0 [Reference]
Holiday	11.11 (1.05) [9.05 - 13.16]	10.59 (30706)		<.001

Equations:

$$Y(AC)_{ij} = \beta_{00} + \beta_{10} * weekend_{ij} + \mu_{ij} + r_{ij}$$
$$Y(AC)_{ij} = \beta_{00} + \beta_{10} * holiday_{ij} + \mu_{ij} + r_{ij}$$

$$Y(AC)_{ij} = \beta_{00} + \beta_{01} * age_j + \beta_{02} * AUDcriteria_j + \beta_{03} * depression_j + \beta_{04} * profession_j + \beta_{05} * graduation_j + \beta_{06} * status_j + \beta_{07} * child_j + \beta_{08} * site_j + \beta_{10} * weekend_{ij} + \mu_{ij} + r_{ij}$$

$$Y(AC)_{ij} = \beta_{00} + \beta_{01} * age_j + \beta_{02} * AUDcriteria_j + \beta_{03} * depression_j + \beta_{04} * profession_j + \beta_{05} * graduation_j + \beta_{06} * status_j + \beta_{07} * child_j + \beta_{08} * site_j + \beta_{10} * holiday_{ij} + \mu_{ij} + r_{ij}$$

eTable 7 Results of moderation analyses gender * weekends/holidays for the outcome AC
In order to examine whether the effect of weekends/holidays AC was different across gender groups multilevel moderation analyses were applied. To control for the influence of covariates the models were extended by the between-subject variables age, AUD criteria, depression, and site. Moreover, to control for overall higher AC among males compared to females, the outcome variable was the mean-centered value of AC.

	**Moderation analyses for gender and weekend/holiday**<br>**Outcome: alcohol consumption** (centered)			
Predictor	β coefficient (SE) [95% CI]	t(df)	F-value	p-value
Weekend				
Intercept	-3.48 (1.47) [-6.36 - -0.60]	-2.37 (48638)	8.02	.018
Female	0 [Reference]	0 [Reference]	0.57	0 [Reference]
Male	-2.34 (0.50) [-3.33 - -1.35]	-4.67 (324)		<.001
Weekday	0 [Reference]	0 [Reference]	1722.66	0 [Reference]
Weekend	11.16 (0.60) [9.99 - 12.34]	18.64 (48638)		<.001
Female * Weekday	0 [Reference]	0 [Reference]	67.75	0 [Reference]
Male * Weekend	6.20 (0.75) [4.73 - 7.68]	8.23 (48638)		<.001
Holiday				
Intercept	0.76 (1.47) [-2.16 - 3.68]	0.52 (48638)	7.75	.604
Female	0 [Reference]	0 [Reference]	0.68	0 [Reference]
Male	0.04 (0.41) [-0.76 - 0.84]	0.11 (324)		.914
No holiday	0 [Reference]	0 [Reference]	131.38	0 [Reference]
Holiday	5.97 (1.27) [3.48 - 8.46]	4.68 (48638)		<.001
Female * no Holiday	0 [Reference]	0 [Reference]	7.87	0 [Reference]
Male * Holiday	4.48 (1.60) [1.31 - 7.65]	2.80 (48638)		.005

Equations:

$$Y(AC\_centered)_{ij} = \beta_{00}+\beta_{01} * age_j + \beta_{02} * gender_j + \beta_{03} * AUDcriteria_j + \beta_{04} * depression_j + \beta_{05} * profession_j + \beta_{06} * graduation_j + \beta_{07} * status_j + \beta_{08} * child_j + \beta_{09} * site_j + \beta_{10} * weekend_{ij} + \beta_{20} * gender_j * weekend_{ij} + \mu_{ij} + r_{ij}$$

$$Y(AC\_centered)_{ij} = \beta_{00}+\beta_{01} * age_j + \beta_{02} * gender_j + \beta_{03} * AUDcriteria_j + \beta_{04} * depression_j + \beta_{05} * profession_j + \beta_{06} * graduation_j + \beta_{07} * status_j + \beta_{08} * child_j + \beta_{09} * site_j + \beta_{10} * holiday_{ij} + \beta_{20} * gender_j * holiday_{ij} + \mu_{ij} + r_{ij}$$

eTable 8 Results of basic multilevel models including only one variable for valence prediction Predictors comprised all within- (level 1) and between-subject (level 2) variables.

	**Basic models Outcome: valence score**			
Predictor	β coefficient (SE) [95% CI]	t(df)	F-value	p-value
Mean Valence	10.35 (0.11) [10.14 - 10.56]	95.69 (50019)	9156.51	<.001
Gender				
Intercept (female)	10.12 (0.18) [9.77 - 10.48]	55.67 (50019)	9194.19	<.001
Male	0.35 (0.23) [-0.09 - 0.80]	1.56 (356)	2.44	<.119
Age				
Intercept (age = 17)	10.80 (0.34) [10.13 - 11.46]	31.78 (50019)	9179.04	<.001
Age	-0.01 (0.01) [-0.03 - 0.01]	-1.37 (356)	1.89	.170
AUD criteria				
Intercept (AUD = 2)	11.85 (0.32) [11.21 - 12.48]	36.59 (50019)	10073.28	<.001
AUD criteria	-0.36 (0.06) [-0.49 - -0.23]	-5.62 (356)	30.81	<.001
Depression diagnosis				
Intercept (no depression)	10.64 (0.12) [10.40 - 10.88]	87.06 (49815)	9642.18	<.001
Depression diagnosis	-1.13 (0.24) [-1.61 - -0.66]	-4.68 (355)	21.93	<.001
Current profession				
Intercept (no job)	10.53 (0.26) [10.02 - 11.04]	40.70 (48176)	8779.71	<.001
Current job	-0.20 (0.29) [-0.76 - 0.36]	-0.70 (343)	0.48	.486
Highest school qualification				
Intercept (no degree)	10.13 (0.61) [8.93 - 11.33]	16.52 (48176)	8965.46	<.001
Pupil	3.51 (2.12) [-0.66 - 7.68]	1.66 (337)		.099
Current career-based training	-	-		-
Secondary general school certificate	-1.01 (1.03) [-3.03 - 1.02]	-0.98 (337)		.329
General Certificate of Secondary Education	0.05 (0.67) [-1.26 - 1.36]	0.07 (337)		.940
Polytechnic secondary school	-0.70 (1.09) [-2.86 - 1.45]	-0.64 (337)	1.96	.520
Advanced technical college certificate	0.74 (0.71) [-0.66 - 2.13]	1.04 (337)		.298
General Certificate of Education	0.30 (0.63) [-0.93 - 1.54]	0.49 (337)		.628
other school degree	-2.00 (1.19) [-4.34 - 0.33]	-1.69 (337)		.092
Marital status				
Intercept (single)	10.43 (0.16) [10.12 - 10.74]	65.46 (48176)	8757.68	<.001
Marriage/partnership	-0.13 (0.23) [-0.59 - 0.33]	-0.57 (340)		.571
Living separately	0.59 (0.57) [-0.53 - 1.72]	1.04 (340)	0.686	.299
Divorced	-0.51 (0.52) [-1.54 - 0.52]	-0.98 (340)		.330
Widowed	-0.57 (1.19) [-2.92 - 1.78]	-0.48 (340)		.634
Having a child				
Intercept (no child)	10.38 (0.14) [10.12 - 10.65]	76.25 (48176)	8768.34	<.001
At least one child	-0.05 (0.23) [-0.51 - 0.41]	-0.20 (343)	0.04	.844
Study center				
Intercept (CI Mannheim)	10.36 (0.19) [9.97 - 10.70]	54.92 (50019)	9268.98	<.001
Charité Berlin	0.49 (0.29) [-0.09 - 1.07]	1.67 (355)		.192

	Basic models<br>Outcome: valence score			
Predictor	β coefficient (SE) [95% CI]	t(df)	F-value	p-value
TU Dresden	-0.21 (0.24) [-0.70 - 0.28]	-0.85 (355)	3.20	.396
Weekend				
Intercept (weekday)	10.31 (0.11) [10.10 - 10.52]	95.07 (50018)	9155.46	<.001
Weekend	0.11 (0.02) [0.07 - 0.14]	6.30 (50018)	39.75	<.001
Holiday				
Intercept (no holiday)	10.35 (0.11) [10.14 - 10.56]	95.64 (50018)	9157.56	<.001
Holiday	0.10 (0.04) [0.03 - 0.17]	2.67 (50018)	7.13	.008
Lockdown phase				
Intercept (pre-lockdown)	10.13 (0.12) [9.89 - 10.36]	85.46 (50015)	9074.62	<.001
Lockdown light 1	0.00 (0.05) [-0.10 - 0.11]	0.06 (50015)		.955
lockdown hard	0.11 (0.05) [0.01 - 0.21]	2.12 (50015)		.034
lockdown light 2	0.15 (0.05) [0.05 - 0.25]	2.86 (50015)	27.46	.004
post-lockdown	0.30 (0.05) [0.21 - 0.41]	6.05 (50015)		<.001

Equation:

$$Y(valence)_{ij} = \beta_{00} + \beta_{01} * predictor_{(i)j} + \mu_{ij} + r_{ij}$$

eTable 9 Results of basic multilevel models including only one variable for calmness prediction
Predictors comprised all within- (level 1) and between-subject (level 2) variables.

	**Basic models<br>Outcome: calmness score**			
Predictor	β coefficient (SE) [95% CI]	t(df)	F-value	p-value
Mean Calmness	9.97 (0.11) [9.75 - 10.20]	87.98 (50007)	839.94	<.001
Gender				
Intercept (female)	9.64 (0.19) [9.26 - 10.01]	50.75 (50007)	7825.33	<.001
Male	0.52 (0.24) [0.06 - 0.98]	2.20 (356)	4.85	.028
Age				
Intercept (age = 17)	10.39 (0.36) [9.70 - 11.09]	29.19 (50007)	7753.08	<.001
Age	-0.01 (0.01) [-0.03 - 0.01]	-1.25 (356)	1.56	.213
AUD criteria				
Intercept (AUD = 2)	11.35 (0.30) [10.75 - 11.94]	37.41 (50007)	8235.43	<.001
AUD criteria	-0.33 (0.07) [-0.47 - -0.20]	-4.86 (356)	23.61	<.001
Depression diagnosis				
Intercept (no depression)	10.26 (0.13) [10.01 - 10.51]	80.03 (49803)	8124.43	<.001
depression diagnosis	-1.15 (0.25) [-1.65 - -0.65]	-4.53 (355)	20.51	<.001
Current profession				
Intercept (no job)	10.26 (0.27) [9.73 - 10.80]	37.66 (48164)	7315.85	<.001
Current job	-0.36 (0.30) [-0.95 - 0.23]	-1.20 (343)	1.43	.233
Highest school qualification				
Intercept (no degree)	9.95 (0.65) [8.68 - 11.22]	15.31 (48164)	7387.46	<.001
Pupil	3.66 (2.24) [-0.76 - 8.07]	1.63 (337)		.104
Current career-based training	-	-		-
Secondary general school certificate	-0.86 (1.09) [-3.01 - 1.29]	-0.79 (337)		.430
General Certificate of Secondary Education	-0.14 (0.71) [-1.53 - 1.25]	-0.20 (337)	1.55	.844
Polytechnic secondary school	-0.61 (1.16) [-2.89 - 1.67]	-0.52 (337)		.601
Advanced technical college certificate	0.62 (0.75) [-0.85 - 2.10]	0.83 (337)		.408
General Certificate of Education	0.03 (0.67) [-1.28 - 1.34]	0.05 (337)		.960
other school degree	-2.03 (1.26) [-4.50 - 0.44]	-1.61 (337)		.107
Marital status				
Intercept (single)	10.08 (0.17) [9.75 - 10.41]	59.97 (48164)	7275.07	<.001
Marriage/partnership	-0.24 (0.25) [-0.73 - 0.24]	-0.98 (340)		.327
Living separately	0.47 (0.60) [-0.72 - 1.66]	0.78 (340)	0.65	.437
Divorced	-0.43 (0.55) [-1.52 - 0.66]	-0.77 (340)		.439
Widowed	-0.75 (1.26) [-3.22 - 1.73]	-0.59 (340)		.555
Having a child				
Intercept (no child)	9.97 (0.14) [9.69 - 10.25]	69.39 (48164)	7285.56	<.001
At least one child	0.00 (0.25) [-0.48 - 0.49]	0.01 (343)	0.00	.999
Study center				
Intercept (CI Mannheim)	9.98 (0.20) [9.59 - 10.37]	50.50 (50007)	7795.84	<.001

	Basic models<br>Outcome: calmness score			
Predictor	β coefficient (SE) [95% CI]	t(df)	F-value	p-value
Charité Berlin	0.40 (0.31) [-0.21 - 1.01]	1.28 (355)	2.26	.199
TU Dresden	-0.22 (0.26) [-0.73 - 0.29]	-0.85 (355)		.394
Weekend				
Intercept (weekday)	9.90 (0.11) [9.68 - 10.12]	87.14 (50006)	7739.75	<.001
Weekend	0.18 (0.02) [0.15 - 0.22]	10.11 (50006)	102.22	<.001
Holiday				
Intercept (no holiday)	9.96 (0.11) [9.74 - 10.19]	87.91 (50006)	7744.87	<.001
Holiday	0.20 (0.04) [0.13 - 0.27]	5.34 (50006)	28.49	<.001
Lockdown phase				
Intercept (pre-lockdown)	9.68 (0.12) [9.44 - 9.92]	77.85 (50003)	7611.70	<.001
Lockdown light 1	-0.04 (0.06) [-0.15 - 0.08]	-0.63 (50003)	51.82	.531
Lockdown hard	0.14 (0.05) [0.03 - 0.24]	2.55 (50003)		.011
Lockdown light 2	0.17 (0.05) [0.06 - 0.27]	3.15 (50003)		.002
Post-lockdown	0.40 (0.05) [0.30 - 0.51]	7.75 (50003)		<.001

Equation:

$$Y(calmness)_{ij} = \beta_{00} + \beta_{01} * predictor_{(i)j} + \mu_{ij} + r_{ij}$$

eTable 10 Results of the main multilevel model for valence prediction
All within-subject (level 1) and between-subject (level 2) variables were included in one model.

	**Main model Outcome: valence score**			
Predictor	β coefficient (SE) [95% CI]	t(df)	F-value	p-value
intercept	11.51 (0.73) [10.08 - 12.93]	15.82 (47966)	10277.23	<.001
Gender				
Female	0 [Reference]	0 [Reference]	4.37	0 [Reference]
Male	0.30 (0.22) [-0.13 - 0.73]	1.37 (324)		.172
Age	-0.02 (0.01) [-0.04 - 0.01]	-1.24 (324)	2.54	.216
AUD criteria	-0.36 (0.07) [-0.48 - -0.23]	-5.38 (324)	35.36	<.001
Depression diagnosis				
No depression	0 [Reference]	0 [Reference]	12.38	0 [Reference]
Depression diagnosis	-0.85 (0.25) [-1.34 - -0.36]	-3.44 (324)		<.001
Current profession				
No current job	0 [Reference]	0 [Reference]	0.76	0 [Reference]
Current job	-0.28 (0.27) [-0.82 - 0.26]	-1.02 (324)		.306
Highest school qualification				
no school degree	0 [Reference]	0 [Reference]	2.09	0 [Reference]
Pupil	3.24 (2.00) [-0.68 - 7.17]	1.63 (324)		.105
Current career-based training	-	-		-
Secondary general school certificate	-0.01 (0.99) [-1.95 - 1.94]	-0.01 (324)		.994
General Certificate of Secondary Education	0.55 (0.66) [-0.74 - 1.85]	0.84 (324)		.401
Polytechnic secondary school	-0.70 (1.08) [-2.82 - 1.41]	-0.65 (324)		.513
Advanced technical college certificate	1.33 (0.70) [-0.05 - 2.70]	1.89 (324)		.059
General Certificate of Education	0.72 (0.62) [-0.49 - 1.94]	1.17 (324)		.242
other school degree	-1.48 (1.16) [-3.77 - 2.70]	-1.27 (324)		.204
Marital status				
Single	0 [Reference]	0 [Reference]	0.81	0 [Reference]
marriage/partnership	-0.12 (0.28) [-0.67 - 0.44]	-0.42 (324)		.676
Living separately	0.77 (0.58) [-0.38 - 1.92]	1.32 (324)		.187
Divorced	-0.15 (0.56) [-1.25 - 0.94]	-0.28 (324)		.781
Widowed	0.33 (1.15) [-1.94 - 2.60]	0.29 (324)		.775
Having a child				
No child	0 [Reference]	0 [Reference]	1.48	0 [Reference]
At least one child	0.27 (0.29) [-0.30 - 0.84]	0.95 (324)		.345
Study center				
CI Mannheim	0 [Reference]	0 [Reference]	3.24	0 [Reference]
Charité Berlin	0.58 (0.29) [0.00 - 1.15]	1.98 (324)		.049
TU Dresden	-0.09 (0.24) [-0.58 - 0.39]	-0.38 (324)		.705
Weekend				
Weekday	0 [Reference]	0 [Reference]	33.68	0 [Reference]
Weekend	0.11 (0.02) [0.07 - 0.14]	5.91 (47966)		<.001

Predictor	Main model<br>Outcome: valence score<br>β coefficient (SE) [95% CI]	t(df)	F-value	p-value
Holiday				
no holiday	0 [Reference]	0 [Reference]	8.00	0 [Reference]
Holiday	0.11 (0.04) [0.03 - 0.18]	2.86 (47966)		.004
Lockdown phase				
Pre-lockdown	0 [Reference]	0 [Reference]	34.69	0 [Reference]
Lockdown light 1	0.01 (0.06) [-0.10 - 0.12]	0.21 (47966)		.836
Lockdown hard	0.12 (0.05) [0.02 - 0.22]	2.37 (47966)		.018
Lockdown light 2	0.16 (0.05) [0.06 - 0.27]	3.12 (47966)		.002
Post-lockdown	0.35 (0.05) [0.25 - 0.45]	6.86 (47966)		<.001

Equation:

$$Y(valence)_{ij} = \beta_{00} + \beta_{01} * age_j + \beta_{02} * gender_j + \beta_{03} * AUDcriteria_j + \beta_{04} * depression_j + \beta_{05} * profession_j + \beta_{06} * graduation_j + \beta_{07} * status_j + \beta_{08} * child_j + \beta_{09} * site_j + \beta_{10} * weekend_{ij} + \beta_{20} * holiday_{ij} + \beta_{30} * lockdown_{ij} + \mu_{ij} + r_{ij}$$

eTable 11 Results of the main multilevel model for calmness prediction
All within-subject (level 1) and between-subject (level 2) variables were included in one model.

	**Main model Outcome: calmness score**			
Predictor	β coefficient (SE) [95% CI]	t(df)	F-value	p-value
intercept	11.18 (0.78) [9.65 - 12.72]	14.30 (47954)	8214.91	<.001
Gender				
Female	0 [Reference]	0 [Reference]	6.48	0 [Reference]
Male	0.42 (0.24) [-0.04 - 0.89]	1.79 (324)		.074
Age	-0.01 (0.01) [-0.04 - 0.01]	-1.03 (324)	2.25	.306
AUD criteria	-0.32 (0.07) [-0.46 - -0.18]	-4.53 (324)	25.32	<.001
Depression diagnosis				
No depression	0 [Reference]	0 [Reference]	12.24	0 [Reference]
Depression diagnosis	-0.93 (0.27) [-1.46 - -0.41]	-3.50 (324)		<.001
Current profession				
No current job	0 [Reference]	0 [Reference]	1.49	0 [Reference]
Current job	-0.44 (0.30) [-1.02 - 0.14]	-1.50 (324)		.134
Highest school qualification				
no school degree	0 [Reference]	0 [Reference]	1.72	0 [Reference]
Pupil	3.46 (2.15) [-0.76 - 7.69]	1.61 (324)		.108
Current career-based training	-	-		-
Secondary general school certificate	0.13 (1.06) [-1.96 - 2.23]	0.12 (324)		.901
General Certificate of Secondary Education	0.39 (0.71) [-1.00 - 1.79]	0.56 (324)		.578
Polytechnic secondary school	-0.62 (1.16) [-2.90 - 1.66]	-0.54 (324)		.591
Advanced technical college certificate	1.23 (0.75) [-0.25 - 2.71]	1.63 (324)		.104
General Certificate of Education	0.47 (0.66) [-0.83 - 1.78]	0.71 (324)		.476
other school degree	-1.51 (1.25) [-3.97 - 0.95]	-1.21 (324)		.229
Marital status				
Single	0 [Reference]	0 [Reference]	0.38	0 [Reference]
marriage/partnership	-0.29 (0.30) [-0.88 - 0.31]	-0.94 (324)		.347
Living separately	0.55 (0.63) [-0.69 - 1.78]	0.87 (324)		.386
Divorced	-0.20 (0.60) [-1.37 - 0.98]	-0.33 (324)		.742
Widowed	0.06 (1.24) [-2.38 - 2.50]	0.05 (324)		.959
Having a child				
No child	0 [Reference]	0 [Reference]	2.15	0 [Reference]
At least one child	0.39 (0.31) [-0.22 - 1.00]	1.26 (324)		.210
Study center				
Mannheim	0 [Reference]	0 [Reference]	2.52	0 [Reference]
Berlin	0.46 (0.31) [-0.16 - 1.07]	1.45 (324)		.149
Dresden	-0.14 (0.27) [-0.67 - 0.38]	-0.54 (324)		.592
Weekend vs weekday				
Weekday	0 [Reference]	0 [Reference]	92.43	0 [Reference]
Weekend	0.18 (0.02) [0.14 - 0.22]	9.68 (47954)		<.001

Predictor	Main model<br>Outcome: calmness score<br>β coefficient (SE) [95% CI]	t(df)	F-value	p-value
Holiday vs no holiday				
no holiday	0 [Reference]	0 [Reference]	25.07	0 [Reference]
Holiday	0.20 (0.04) [0.12 - 0.28]	5.07 (47954)		<.001
Lockdown phase				
Pre-lockdown	0 [Reference]	0 [Reference]	57.35	0 [Reference]
Lockdown light 1	-0.03 (0.06) [-0.15 - 0.08]	-0.56 (47954)		.578
Lockdown hard	0.13 (0.05) [0.02 - 0.24]	2.41 (47954)		.016
Lockdown light 2	0.18 (0.05) [0.07 - 0.28]	3.22 (47954)		.001
Post-lockdown	0.44 (0.05) [0.33 - 0.54]	8.14 (47954)		<.001

Equation:

$$Y(calmness)_{ij} = \beta_{00} + \beta_{01} * age_j + \beta_{02} * gender_j + \beta_{03} * AUDcriteria_j + \beta_{04} * depression_j + \beta_{05} * profession_j + \beta_{06} * graduation_j + \beta_{07} * status_j + \beta_{08} * child_j + \beta_{09} * site_j + \beta_{10} * weekend_{ij} + \beta_{20} * holiday_{ij} + \beta_{30} * lockdown_{ij} + \mu_{ij} + r_{ij}$$

eTable 12 Results of moderation analyses gender * lockdown phase for the outcome mood

In order to examine whether the effect of lockdown phase on valence and calmness was different across gender groups multilevel moderation analyses were applied. To control for the influence of covariates the models were extended by the between- and within-subject variables age, AUD criteria, depression, site, weekend, and holiday.

	**Moderation analyses for gender and lockdown phase Outcome: mood score (valence or calmness)**			
Predictor	β coefficient (SE) [95% CI]	t(df)	F-value	p-value
**Valence**				
Intercept	11.74 (0.73) [9.96 - 13.03]	16.04 (47962)	10228.23	<.001
Female * pre-lockdown	0 [Reference]	0 [Reference]		
Male * lockdown light 1	-0.07 (0.12) [-0.12 - 0.35]	-0.59 (47962)	19.42	.556
Male * lockdown hard	0.27 (0.11) [0.17 - 0.62]	2.48 (47962)		.013
Male * lockdown light 2	0.58 (0.11) [0.47 - 0.91]	5.31 (47962)		<.001
Male * post-lockdown	0.34 (0.11) [0.22 - 0.66]	3.12 (47962)		.002
**Calmness**				
Intercept	11.49 (0.79) [10.31 - 12.38]	14.61 (47950)	8183.99	<.001
Female * pre-lockdown	0 [Reference]	0 [Reference]		
Male * lockdown light 1	0.11 (0.12) [-0.12 - 0.35]	0.94 (47950)	17.13	.346
Male * lockdown hard	0.40 (0.11) [0.17 - 0.62]	3.51 (47950)		<.001
Male * lockdown light 2	0.69 (0.11) [0.47 - 0.92]	6.07 (47950)		<.001
Male * post-lockdown	0.44 (0.11) [0.22 - 0.66]	3.93 (47950)		<.001

Equations:

$$Y(valence)_{ij} = \beta_{00} + \beta_{01} * age_j + \beta_{02} * gender_j + \beta_{03} * AUDcriteria_j + \beta_{04} * depression_j + \beta_{05} * profession_j + \beta_{06} * graduation_j + \beta_{07} * status_j + \beta_{08} * site_j + \beta_{10} * weekend_{ij} + \beta_{20} * holiday_{ij} + \beta_{30} * lockdown_{ij} + \beta_{40} * gender_j * lockdown_{ij} + \mu_{ij} + r_{ij}$$

$$Y(calmness)_{ij} = \beta_{00} + \beta_{01} * age_j + \beta_{02} * gender_j + \beta_{03} * AUDcriteria_j + \beta_{04} * depression_j + \beta_{05} * profession_j + \beta_{06} * graduation_j + \beta_{07} * status_j + \beta_{08} * site_j + \beta_{10} * weekend_{ij} + \beta_{20} * holiday_{ij} + \beta_{30} * lockdown_{ij} + \beta_{40} * gender_j * lockdown_{ij} + \mu_{ij} + r_{ij}$$

eTable 13 Results of multilevel models examining the gender-specific influence of lockdown phases on valence and calmness

To control for the influence of covariates the models were extended by the between- and within-subject variables age, AUD criteria, depression, site, weekend, and holiday.

	**Gender-specific lockdown models Outcome: mood score (valence or calmness)**			
Predictor	β coefficient (SE) [95% CI]	t(df)	F-value	p-value
**Females (N = 126)**				
Valence				
Intercept (pre-lockdown)	12.27 (1.28) [9.76 - 14.78]	9.58 (17682)	3304.60	<.001
lockdown light 1	0.06 (0.10) [-0.15 - 0.27]	0.57 (17682)	14.07	.567
lockdown hard	-0.05 (0.10) [-0.24 - 0.14]	-0.52 (17682)		.603
lockdown light 2	-0.20 (0.10) [-0.40 - 0.01]	-2.06 (17682)		.039
post-lockdown	0.14 (0.10) [-0.05 - 0.33]	1.41 (17682)		.158
Calmness				
Intercept (pre-lockdown)	12.21 (1.38) [9.51 - 14.91]	8.56 (17678)	2578.96	<.001
lockdown light 1	-0.10 (0.11) [-0.32 - 0.12]	-0.91 (17678)	19.20	.362
lockdown hard	-0.13 (0.10) [-0.33 - 0.07]	-1.26 (17678)		.208
lockdown light 2	-0.27 (0.10) [-0.47 - -0.06]	-2.58 (17678)		.010
post-lockdown	0.15 (0.10) [-0.05 - 0.35]	1.49 (17678)		.135
**Males (N = 232)**				
Valence				
Intercept (pre-lockdown)	11.37 (0.90) [9.60 - 13.15]	12.57 (30278)	6745.25	<.001
lockdown light 1	-0.01 (0.06) [-0.14 - 0.11]	-0.16 (30278)	41.37	.873
lockdown hard	0.21 (0.06) [0.10 - 0.33]	3.57 (30278)		<.001
lockdown light 2	0.37 (0.06) [0.25 - 0.49]	6.14 (30278)		<.001
post-lockdown	0.47 (0.06) [0.35 - 0.59]	7.88 (30278)		<.001
Calmness				
Intercept (pre-lockdown)	10.99 (0.97) [9.10 - 12.89]	11.36 (30270)	5511.34	<.001
lockdown light 1	0.01 (0.07) [-0.12 - 0.14]	0.12 (30270)	57.18	.904
lockdown hard	0.27 (0.06) [0.15 - 0.39]	4.31 (30270)		<.001
lockdown light 2	0.42 (0.06) [0.30 - 0.55]	6.78 (30270)		<.001
post-lockdown	0.59 (0.06) [0.47 - 0.71]	9.55 (30270)		<.001

Equations:

$$Y(valence)_{ij} = \beta_{00} + \beta_{01} * age_j + \beta_{02} * AUDcriteria_j + \beta_{03} * depression_j + \beta_{04} * profession_j + \beta_{05} * graduation_j + \beta_{06} * status_j + \beta_{07} * site_j + \beta_{10} * weekend_{ij} + \beta_{20} * holiday_{ij} + \beta_{30} * lockdown_{ij} + \mu_{ij} + r_{ij}$$

$$Y(calmness)_{ij} = \beta_{00} + \beta_{01} * age_j + \beta_{02} * AUDcriteria_j + \beta_{03} * depression_j + \beta_{04} * profession_j + \beta_{05} * graduation_j + \beta_{06} * status_j + \beta_{07} * site_j + \beta_{10} * weekend_{ij} + \beta_{20} * holiday_{ij} + \beta_{30} * lockdown_{ij} + \mu_{ij} + r_{ij}$$

eTable 14 Results of basic multilevel models including only one variable for PSS prediction Predictors comprised all within- (level 1) and between-subject (level 2) variables.

	**Basic models Outcome: perceived stress score**			
Predictor	β coefficient (SE) [95% CI]	t(df)	F-value	p-value
Mean PSS score	15.85 (0.36) [15.19 - 16.51]	47.22 (46119)	2230.17	<.001
Gender				
Intercept (female)	17.64 (0.55) [16.57 - 18.72]	32.04 (46119)	2335.72	<.001
Male	-2.78 (0.68) [-4.13 - -1.43]	-4.06 (328)	16.47	<.001
Age				
Intercept (age = 17)	18.04 (1.07) [15.94 - 20.14]	16.85 (46119)	2255.13	<.001
Age	-0.06 (0.03) [-0.11 - -0.00]	-2.15 (328)	4.64	.032
AUD criteria				
Intercept (AUD = 2)	12.04 (0.92) [10.24 - 13.85]	13.07 (46119)	2355.08	<.001
Slope (AUD criteria)	0.92 (0.21) [0.51 - 1.33]	4.41 (328)	19.49	<.001
Depression diagnosis				
Intercept (no depression)	15.24 (0.39) [14.49 - 16.00]	39.50 (45909)	2271.60	<.001
Depression diagnosis	2.30 (0.76) [0.81 - 3.80]	3.03 (327)	9.20	.003
Current profession				
Intercept (no job)	15.22 (0.81) [13.63 - 16.80]	18.81 (44781)	2120.67	<.001
Current job	0.82 (0.89) [-0.94 - 2.58]	0.92 (317)	0.85	.358
Highest school qualification				
Intercept (no degree)	17.00 (2.01) [13.06 - 20.95]	8.45 (44781)	2210.56	<.001
Pupil	-6.55 (6.35) [-19.06 - 5.95]	-1.03 (311)		.303
Current career-based training	-	-		-
Secondary general school certificate	4.06 (3.18) [-2.20 - 10.32]	1.28 (311)		.203
General Certificate of Secondary Education	1.17 (2.17) [-3.10 - 5.44]	0.54 (311)		.590
Polytechnic secondary school	0.63 (3.37) [-5.99 - 7.26]	0.19 (311)	2.90	.851
Advanced technical college certificate	-2.69 (2.29) [-7.19 - 1.81]	-1.18 (311)		.240
General Certificate of Education	-1.76 (2.06) [-5.80 - 2.28]	-0.86 (311)		.393
other school degree	3.75 (4.02) [-4.16 - 11.66]	0.93 (311)		.352
Marital status				
Intercept (single)	16.42 (0.50) [15.43 - 17.41]	32.54 (44781)	2154.27	<.001
Marriage/partnership	-0.66 (0.72) [-2.09 - 0.76]	-0.91 (314)		.362
Living separately	-4.91 (1.71) [-8.27 - -1.54]	-2.87 (314)	2.21	.004
Divorced	-0.82 (1.57) [-3.90 - 2.27]	-0.52 (314)		.603
Widowed	1.85 (3.56) [-5.16 - 8.87]	0.52 (314)		.604
Having a child				
Intercept (no child)	16.26 (0.43) [15.42 - 17.09]	38.01 (44781)	2128.85	<.001
At least one child	-1.03 (0.72) [-2.45 - 0.39]	-1.43 (317)	2.05	.153
Study center				
Intercept (CI Mannheim)	15.45 (0.58) [14.32 - 16.59]	26.72 (46119)	2231.25	<.001
Charité Berlin	-0.12 (0.92) [-1.94 - 1.69]	-0.13 (327)		.895

	**Basic models**<br>**Outcome: perceived stress score**			
Predictor	β coefficient (SE) [95% CI]	t(df)	F-value	p-value
TU Dresden	0.94 (0.77) [-0.56 - 2.45]	1.23 (327)	1.09	.219
Weekend				
Intercept (weekday)	15.85 (0.34) [15.19 - 16.51]	47.19 (46118)	2230.17	<.001
Weekend	0.00 (0.03) [-0.06 - 0.06]	0.00 (46118)	0.00	.998
Holiday				
Intercept (no holiday)	15.83 (0.34) [15.18 - 16.49]	47.17 (46118)	2229.66	<.001
Holiday	0.29 (0.06) [0.17 - 0.42]	4.67 (46118)	21.80	<.001
Lockdown phase				
Intercept (pre-lockdown)	16.44 (0.35) [15.76 - 17.12]	47.44 (46115)	2199.99	<.001
Lockdown light 1	0.09 (0.09) [-0.10 - 0.27]	0.92 (46115)		.358
Lockdown hard	0.32 (0.09) [0.16 - 0.50]	3.78 (46115)		<.001
Lockdown light 2	0.25 (0.09) [0.08 - 0.41]	2.91 (46115)	332.72	.004
Post-lockdown	-1.11 (0.08) [-1.27 - -0.94]	-13.15 (46115)		<.001

Equation:

$$Y(PSS)_{ij} = \beta_{00} + \beta_{01} * predictor_j + \mu_{ij} + r_{ij}$$

eTable 15 Results of the main multilevel model for perceived stress as the outcome variable
All within-subject (level 1) and between-subject (level 2) variables were included in one model.

	**Main model Outcome: perceived stress score**			
Predictor	β coefficient (SE) [95% CI]	t(df)	F-value	p-value
intercept	15.89 (2.41) [11.17 - 20.61]	6.60 (44567)	2469.25	<.001
Gender				
Female	0 [Reference]	0 [Reference]	17.62	0 [Reference]
Male	-2.24 (0.69) [-3.59 - -0.88]	-3.25 (298)		<.001
Age	-0.06 (0.04) [-0.14 - 0.01]	-1.60 (298)	3.90	.110
AUD criteria	0.81 (0.21) [0.40 - 1.23]	3.83 (298)	18.26	<.001
Depression diagnosis				
No depression	0 [Reference]	0 [Reference]	5.54	0 [Reference]
Depression diagnosis	1.77 (0.77) [0.25 - 3.30]	2.29 (298)		.023
Current profession				
No current job	0 [Reference]	0 [Reference]	1.17	0 [Reference]
Current job	0.86 (0.86) [-0.83 - 2.55]	1.00 (298)		.317
Highest school qualification				
no school degree	0 [Reference]	0 [Reference]	2.78	0 [Reference]
Pupil	-4.59 (6.07) [-16.54 - 7.36]	-0.76 (298)		.450
Current career-based training	-	-		-
Secondary general school certificate	3.32 (3.09) [-2.75 - 9.39]	1.08 (298)		.283
General Certificate of Secondary Education	1.80 (2.16) [-2.44 - 6.04]	0.84 (298)		.404
Polytechnic secondary school	3.90 (3.34) [-2.67 - 10.47]	1.17 (298)		.244
Advanced technical college certificate	-1.93 (2.27) [-6.40 - 2.55]	-0.85 (298)		.397
General Certificate of Education	-0.71 (2.03) [-4.70 - 3.28]	-0.35 (298)		.725
other school degree	6.12 (3.98) [-1.71 - 13.96]	1.54 (298)		.125
Marital status				
Single	0 [Reference]	0 [Reference]	1.69	0 [Reference]
marriage/partnership	0.61 (0.88) [-1.12 - 2.35]	0.70 (298)		.486
Living separately	-3.32 (1.77) [-6.81 - 0.17]	-1.87 (298)		.063
Divorced	0.14 (1.69) [-3.18 - 3.46]	0.08 (298)		.935
Widowed	1.51 (3.49) [-5.36 - 8.37]	0.43 (298)		.667
Having a child				
No child	0 [Reference]	0 [Reference]	0.54	0 [Reference]
At least one child	-0.50 (0.89) [-2.25 - 1.26]	-0.56 (298)		.577
Study center				
CI Mannheim	0 [Reference]	0 [Reference]	1.83	0 [Reference]
Charité Berlin	-1.04 (0.93) [-2.87 - 0.78]	-1.12 (298)		.262
TU Dresden	0.65 (0.78) [-0.89 - 2.19]	0.83 (298)		.405
Lockdown phase				
Pre-lockdown	0 [Reference]	0 [Reference]		0 [Reference]
Lockdown light 1	0.04 (0.09) [-0.15 - 0.22]	0.40 (44567)		.690

	Main model<br>Outcome: perceived stress score			
Predictor	β coefficient (SE) [95% CI]	t(df)	F-value	p-value
Lockdown hard	0.38 (0.09) [0.21 - 0.55]	4.30 (44567)	312.93	<.001
Lockdown light 2	0.24 (0.09) [0.07 - 0.41]	2.73 (44567)		.006
Post-lockdown	-1.09 (0.09) [-1.26 - -0.92]	-12.75 (44567)		<.001

Equation:

$$Y(perceived\ stress)_{ij} = \beta_{00} + \beta_{01} * age_j + \beta_{02} * gender_j + \beta_{03} * AUDcriteria_j + \beta_{04} * depression_j + \beta_{05} * profession_j + \beta_{06} * graduation_j + \beta_{07} * status_j + \beta_{08} * child_j + \beta_{09} * site_j + \beta_{10} * lockdown_{ij} + \mu_{ij} + r_{ij}$$

eTable 16 Results of moderation analyses gender * lockdown phase for the outcome PSS
In order to examine whether the effect of lockdown phase on perceived stress was different across gender groups multilevel moderation analyses were applied. To control for the influence of covariates the models were extended by the between-subject variables age, AUD criteria, depression, and site.

	**Moderation analyses for gender and lockdown phase Outcome: perceived stress score**			
Predictor	β coefficient (SE) [95% CI]	t(df)	F-value	p-value
**Perceived stress**				
Intercept	15.74 (2.41) [11.01 - 20.46]	6.53 (44563)	2468.26	<.001
Female * pre-lockdown	0 [Reference]	0 [Reference]		0 [Reference]
Male * lockdown light 1	-0.78 (0.20) [-1.16 - -0.39]	-3.96 (44563)		<.001
Male * lockdown hard	0.22 (0.18) [-0.13 - 0.58]	1.22 (44563)	14.77	.223
Male * lockdown light 2	-0.41 (0.18) [-0.76 - -0.06]	-2.30 (44563)		.021
Male * post-lockdown	-0.14 (0.18) [-0.48 - 0.21]	-0.78 (44563)		.433

Equation:

$$Y(PSS)_{ij} = \beta_{00} + \beta_{01} * age_j + \beta_{02} * gender_j + \beta_{03} * AUDcriteria_j + \beta_{04} * depression_j + \beta_{05} * profession_j + \beta_{06} * graduation_j + \beta_{07} * status_j + \beta_{08} * child_j + \beta_{09} * site_j + \beta_{10} * lockdown_{ij} + \beta_{20} * gender_j * lockdown_{ij} + \mu_{ij} + r_{ij}$$

eTable 17 Results of multilevel models examining the gender-specific influence of lockdown phases on PSS

To control for the influence of covariates the models were extended by the between- and within-subject variables age, AUD criteria, depression, and site.

	**Gender-specific lockdown models**<br>**Outcome: perceived stress score**			
Predictor	β coefficient (SE) [95% CI]	t(df)	F-value	p-value
**Females (N = 126)**				
Intercept (pre-lockdown)	16.42 (4.43) [7.74 - 25.11]	3.71 (16687)	1028.52	<.001
lockdown light 1	0.54 (0.17) [0.20 - 0.87]	3.15 (16687)	115.46	.002
lockdown hard	0.23 (0.16) [-0.08 - 0.54]	1.45 (16687)		.147
lockdown light 2	0.49 (0.15) [0.19 - 0.79]	3.18 (16687)		.002
post-lockdown	-1.01 (0.15) [-1.30 - -0.71]	-6.67 (16687)		<.001
**Males (N = 232)**				
Intercept (pre-lockdown)	13.03 (2.93) [7.28 - 18.78]	4.44 (27876)	1395.64	<.001
lockdown light 1	-0.24 (0.11) [-0.46 - -0.02]	-2.15 (27876)	214.59	.031
lockdown hard	0.45 (0.10) [0.25 - 0.66]	4.32 (27876)		<.001
lockdown light 2	0.08 (0.10) [-0.13 - 0.28]	0.72 (27876)		.469
post-lockdown	-1.15 (0.10) [-1.35 - -0.95]	-11.17 (27876)		<.001

Equation:

$$Y(PSS)_{ij} = \beta_{00} + \beta_{01} * age_j + \beta_{02} * AUDcriteria_j + \beta_{03} * depression_j + \beta_{04} * profession_j + \beta_{05} * graduation_j + \beta_{06} * status_j + \beta_{07} * child_j + \beta_{08} * site_j + \beta_{10} * lockdown_{ij} + \mu_{ij} + r_{ij}$$

eTable 18 Results of multilevel models for the AC prediction by valence scores
The valence values were mean-centered to control for between-person effects. To control for the influence of other between- and within-subjects variables (in particular weekends and holidays which were found to be significantly associated with mood and AC), the original basic model was successively extended by covariates.

	**Multilevel models for AC prediction by valence scores<br>Outcome: alcohol consumption**			
Predictor	β coefficient (SE) [95% CI]	t(df)	F-value	p-value
**Valence**				
no covariates				
Intercept	36.63 (1.29)	28.49 (46039)	810.15	<.001
Valence_c	0.57 (0.20) [0.18 - 0.96]	2.90 (46039)	8.40	.004
including between- and within-subject variables (without weekend and holiday)				
Intercept	4.47 (8.81)	0.51 (44224)	873.85	.612
Valence_c	0.53 (0.20) [0.13 - 0.92]	2.58 (44224)	7.28	.010
**Controlling for the influence of weekend and holiday**				
including between- and within-subject variables (without weekend)				
Intercept	4.48 (8.81)	0.51 (44223)	874.95	.611
Valence_c	0.51 (0.20) [0.11 - 0.90]	2.50 (44223)	7.33	.012
including between- and within-subject variables (without holiday)				
Intercept	-2.10 (8.78)	-0.24 (44223)	877.59	.811
Valence_c	0.42 (0.20) [0.03 - 0.81]	2.11 (44223)	7.69	.035
including all between- and within-subject variables				
Intercept	-2.02 (8.78)	-0.23 (44222)	878.49	.818
Valence_c	0.40 (0.20) [0.02 - 0.79]	2.04 (44222)	7.73	.041

Equations:

$$Y(AC)_{ij} = \beta_{00} + \beta_{01} * valence\_centered_{ij} + \mu_{ij} + r_{ij}$$

$$Y(AC)_{ij} = \beta_{00} + \beta_{01} * age_j + \beta_{02} * gender_j + \beta_{03} * AUDcriteria_j + \beta_{04} * depression_j + \beta_{05} * profession_j + \beta_{06} * graduation_j + \beta_{07} * status_j + \beta_{08} * child_j + \beta_{09} * site_j + \beta_{10} * lockdown_{ij} + \beta_{20} * valence\_centered_{ij} + \mu_{ij} + r_{ij}$$

Controlling for the influence of weekend and holiday

$$Y(AC)_{ij} = \beta_{00} + \beta_{01} * age_j + \beta_{02} * gender_j + \beta_{03} * AUDcriteria_j + \beta_{04} * depression_j + \beta_{05} * profession_j + \beta_{06} * graduation_j + \beta_{07} * status_j + \beta_{08} * child_j + \beta_{09} * site_j + \beta_{10} * lockdown_{ij} + \beta_{20} * valence\_centered_{ij} + \beta_{30} * holiday_{ij} + \mu_{ij} + r_{ij}$$

$$Y(AC)_{ij} = \beta_{00} + \beta_{01} * age_j + \beta_{02} * gender_j + \beta_{03} * AUDcriteria_j + \beta_{04} * depression_j + \beta_{05} * profession_j + \beta_{06} * graduation_j + \beta_{07} * status_j + \beta_{08} * child_j + \beta_{09} * site_j + \beta_{10} * lockdown_{ij} + \beta_{20} * valence\_centered_{ij} + \beta_{30} * weekend_{ij} + \mu_{ij} + r_{ij}$$

$$Y(AC)_{ij} = \beta_{00} + \beta_{01} * age_j + \beta_{02} * gender_j + \beta_{03} * AUDcriteria_j + \beta_{04} * depression_j + \beta_{05} * profession_j + \beta_{06} * graduation_j + \beta_{07} * status_j + \beta_{08} * child_j + \beta_{09} * site_j + \beta_{10} * lockdown_{ij} + \beta_{20} * valence\_centered_{ij} + \beta_{30} * weekend_{ij} + \beta_{40} * holiday_{ij} + \mu_{ij} + r_{ij}$$

eTable 19 Results of multilevel models for the AC prediction by calmness scores

The calmness values were mean-centered to control for between-person effects. To control for the influence of other between- and within-subjects variables (in particular weekends and holidays which were found to be significantly associated with mood and AC), the original basic model was successively extended by covariates.

	**Multilevel models for AC prediction by calmness scores<br>Outcome: alcohol consumption**			
Predictor	β coefficient (SE) [95% CI]	t(df)	F-value	p-value
**Calmness**				
no covariates				
Intercept	36.64 (1.28)	28.58 (46028)	812.45	<.001
Calmness_c	0.42 (0.17) [0.09 - 0.75]	2.47 (46028)	6.12	.013
including between- and within-subject variables (without weekend and holiday)				
Intercept	4.19 (8.79)	0.48 (44213)	879.04	.634
Calmness_c	0.33 (0.17) [0.00 - 0.67]	1.98 (44213)	4.53	.047
**Controlling for the influence of weekend and holiday**				
including between- and within-subject variables (without weekend)				
Intercept	4.21 (8.79)	0.48 (44212)	880.29	.632
Calmness_c	0.31 (0.17) [-0.02 - 0.64]	1.84 (44212)	4.58	.065
including between- and within-subject variables (without holiday)				
Intercept	-2.36 (8.77)	-0.27 (44212)	883.19	.788
Calmness_c	0.17 (0.16) [-0.15 - 0.49]	1.04 (44212)	4.77	.297
including all between- and within-subject variables				
Intercept	-2.28 (8.76)	-0.26 (44211)	884.15	.795
Calmness_c	0.15 (0.16) [-0.17 - 0.47]	0.92 (44211)	4.81	.359

Equations:

$$Y(AC)_{ij} = \beta_{00} + \beta_{01} * calmness\_centered_{ij} + \mu_{ij} + r_{ij}$$

$$Y(AC)_{ij} = \beta_{00} + \beta_{01} * age_j + \beta_{02} * gender_j + \beta_{03} * AUDcriteria_j + \beta_{04} * depression_j + \beta_{05} * profession_j + \beta_{06} * graduation_j + \beta_{07} * status_j + \beta_{08} * child_j + \beta_{09} * site_j + \beta_{10} * lockdown_{ij} + \beta_{20} * calmness\_centered_{ij} + \mu_{ij} + r_{ij}$$

Controlling for the influence of weekend and holiday

$$Y(AC)_{ij} = \beta_{00} + \beta_{01} * age_j + \beta_{02} * gender_j + \beta_{03} * AUDcriteria_j + \beta_{04} * depression_j + \beta_{05} * profession_j + \beta_{06} * graduation_j + \beta_{07} * status_j + \beta_{08} * child_j + \beta_{09} * site_j + \beta_{10} * lockdown_{ij} + \beta_{20} * calmness\_centered_{ij} + \beta_{30} * holiday_{ij} + \mu_{ij} + r_{ij}$$

$$Y(AC)_{ij} = \beta_{00} + \beta_{01} * age_j + \beta_{02} * gender_j + \beta_{03} * AUDcriteria_j + \beta_{04} * depression_j + \beta_{05} * profession_j + \beta_{06} * graduation_j + \beta_{07} * status_j + \beta_{08} * child_j + \beta_{09} * site_j + \beta_{10} * lockdown_{ij} + \beta_{20} * calmness\_centered_{ij} + \beta_{30} * weekend_{ij} + \mu_{ij} + r_{ij}$$

$$Y(AC)_{ij} = \beta_{00} + \beta_{01} * age_j + \beta_{02} * gender_j + \beta_{03} * AUDcriteria_j + \beta_{04} * depression_j + \beta_{05} * profession_j + \beta_{06} * graduation_j + \beta_{07} * status_j + \beta_{08} * child_j + \beta_{09} * site_j + \beta_{10} * lockdown_{ij} + \beta_{20} * calmness\_centered_{ij} + \beta_{30} * weekend_{ij} + \beta_{40} * holiday_{ij} + \mu_{ij} + r_{ij}$$

eTable 20 Results of moderation analyses focusing on the influence of gender in AC prediction

In order to examine whether the prediction of AC by mean and mean-centered values of valence, calmness or PSS scores differed systematically across gender groups multilevel moderation analyses were applied.

	Gender-focused moderation analyses Outcome: alcohol consumption			
Predictor	β coefficient (SE) [95% CI]	t(df)	F-value	p-value
**Valence * gender**				
Intercept	49.56 (11.11)	4.46 (46038)	876.43	<.001
Female * Valence_m	0 [Reference]	0 [Reference]	0.05	0 [Reference]
Male * Valence_m	0.30 (1.31) [-2.28 - 2.88]	0.23 (354)		.820
Female * Valence_c	0 [Reference]	0 [Reference]	0.47	0 [Reference]
Male * Valence_c	0.28 (0.40) [-0.51 - 1.07]	0.69 (46038)		.493
**Calmness * gender**				
Intercept	42.04 (10.10)	4.16 (46027)	873.81	<.001
Female * Calmness_m	0 [Reference]	0 [Reference]	0.03	0 [Reference]
Male * Calmness_m	-0.20 (1.25) [-2.66 - 2.25]	-0.16 (354)		.870
Female * Calmness_c	0 [Reference]	0 [Reference]	3.04	0 [Reference]
Male * Calmness_c	0.60 (0.34) [-0.07 - 1.27]	1.74 (46027)		.081
**Perceived stress * gender**				
Intercept	15.13 (7.31)	2.07 (35058)	819.39	<.001
Female * PSS_m	0 [Reference]	0 [Reference]	1.36	0 [Reference]
Male * PSS_m	-0.56 (0.48) [-1.50 - 0.38]	-1.16 (324)		.247
Female * PSS_c	0 [Reference]	0 [Reference]	3.34	0 [Reference]
Male * PSS_c	-0.15 (0.24) [-0.62 - 0.33]	-0.60 (35058)		.550

Equations:

$$Y(AC)_{ij} = \beta_{00} + \beta_{01} * gender_j + \beta_{02} * valence\_mean_j + \beta_{10} * valence\_centered_{ij} + \beta_{20} * gender_j * valence\_mean_j + \beta_{30} * gender_j * valence\_centered_{ij} + \mu_{ij} + r_{ij}$$

$$Y(AC)_{ij} = \beta_{00} + \beta_{01} * gender_j + \beta_{02} * calmness\_mean_j + \beta_{10} * calmness\_centered_{ij} + \beta_{20} * gender_j * calmness\_mean_j + \beta_{30} * gender_j * calmness\_centered_{ij} + \mu_{ij} + r_{ij}$$

$$Y(AC)_{ij} = \beta_{00} + \beta_{01} * gender_j + \beta_{02} * perceivedstress\_mean_j + \beta_{10} * perceivedstress\_centered_{ij} + \beta_{20} * gender_j * perceivedstress\_mean_j + \beta_{30} * gender_j * perceivedstress\_centered_{ij} + \mu_{ij} + r_{ij}$$

eTable 21 Results of moderation analyses focusing on the influence of lockdown phase in AC prediction

In order to examine whether the prediction of AC by mean and mean-centered values of valence, calmness or PSS scores differed systematically across lockdown phases multilevel moderation analyses were applied.

	**Lockdown-focused moderation analyses Outcome: alcohol consumption**			
Predictor	β coefficient (SE) [95% CI]	t(df)	F-value	p-value
**Valence * lockdown**				
Intercept	49.00 (8.36)	5.86 (46027)	820.06	<.001
Pre-lockdown * Valence_m	0 [Reference]	0 [Reference]		0 [Reference]
lockdown light 1 * Valence_m	-0.71 (0.55) [-1.79 - 0.37]	-1.29 (46027)		.197
lockdown hard * Valence_m	-0.91 (0.52) [-1.93 - 0.11]	-1.75 (46027)	2.53	.079
lockdown light 2 * Valence_m	-0.15 (0.53) [-1.18 - 0.88]	-0.29 (46027)		.770
Post-lockdown * Valence_m	-0.80 (0.52) [-1.82 - 0.21]	-1.55 (46027)		.121
Pre-lockdown * Valence_c	0 [Reference]	0 [Reference]		0 [Reference]
lockdown light 1 * Valence_c	0.25 (0.62) [-0.97 - 1.47]	0.41 (46027)		.685
lockdown hard * Valence_c	-0.10 (0.58) [-1.25 - 1.04]	-0.18 (46027)	4.22	.859
lockdown light 2 * Valence_c	-1.05 (0.58) [-2.19 - 0.08]	-1.81 (46027)		.070
Post-lockdown * Valence_c	-0.01 (0.56) [-1.11 - 1.08]	-0.02 (46027)		.984
**Calmness *lockdown**				
Intercept	46.28 (7.76)	5.96 (46016)	816.97	<.001
Pre-lockdown * Calmness_m	0 [Reference]	0 [Reference]		0 [Reference]
lockdown light 1 * Calmness_m	-0.71 (0.53) [-1.74 - 0.33]	-1.34 (46016)		.182
lockdown hard * Calmness_m	-0.64 (0.50) [-1.62 - 0.34]	-1.28 (46016)	2.48	.200
lockdown light 2 * Calmness_m	0.02 (0.51) [-0.97 - 1.01]	0.03 (46016)		.973
Post-lockdown * Calmness_m	-0.66 (0.50) [-1.64 - 0.32]	-1.32 (46016)		.187
Pre-lockdown * Calmness_c	0 [Reference]	0 [Reference]		0 [Reference]
lockdown light 1 * Calmness_c	-0.43 (0.59) [-1.59 - 0.73]	-0.73 (46016)		.466
lockdown hard * Calmness_c	-0.33 (0.55) [-1.40 - 0.74]	-0.60 (46016)	2.21	.547
lockdown light 2 * Calmness_c	-0.83 (0.54) [-1.90 - 0.24]	-1.53 (46016)		.127
Post-lockdown * Calmness_c	-0.07 (0.52) [-1.09 - 0.95]	-0.13 (46016)		.894
**Perceived stress * lockdown**				
Intercept	34.27 (5.27)	6.50 (35047)	764.38	<.001
Pre-lockdown * PSS_m	0 [Reference]	0 [Reference]		0 [Reference]
lockdown light 1 * PSS_m	-0.02 (0.25) [-0.51 - 0.47]	-0.08 (35047)		.938
lockdown hard * PSS_m	-0.22 (0.24) [-0.69 - 0.24]	-0.94 (35047)	1.76	.345
lockdown light 2 * PSS_m	-0.41 (0.24) [-0.88 - 0.05]	-1.74 (35047)		.082
Post-lockdown * PSS_m	-0.22 (0.24) [-0.68 - 0.24]	-0.95 (35047)		.344
Pre-lockdown * PSS_c	0 [Reference]	0 [Reference]		0 [Reference]
lockdown light 1 * PSS_c	-0.30 (0.41) [-1.10 - 0.50]	-0.73 (35047)		.464
lockdown hard * PSS_c	0.82 (0.40) [0.03 - 1.61]	2.04 (35047)	5.15	.062
lockdown light 2 * PSS_c	0.83 (0.40) [0.05 - 1.60]	2.09 (35047)		.067
Post-lockdown * PSS_c	0.34 (0.39) [-0.42 - 1.10]	0.87 (35047)		.382

Equations:

$$Y(AC)_{ij} = \beta_{00} + \beta_{10} * lockdown_{ij} + \beta_{20} * valence\_centered_{ij} + \beta_{30} * lockdown_{ij} * valence\_centered_{ij} + \mu_{ij} + r_{ij}$$

$$Y(AC)_{ij} = \beta_{00} + \beta_{10} * lockdown_{ij} + \beta_{20} * calmness\_centered_{ij} + \beta_{30} * lockdown_{ij} * calmness\_centered_{ij} + \mu_{ij} + r_{ij}$$

$$Y(AC)_{ij} = \beta_{00} + \beta_{10} * lockdown_{ij} + \beta_{20} * perceivedstress\_centered_{ij} + \beta_{30} * lockdown_{ij} * perceivedstress\_centered_{ij} + \mu_{ij} + r_{ij}$$

eTable 22 Results of disentangling within- and between-person associations for AC prediction
To investigate within- and between-person associations with AC as outcome variable in more detail both, subject-specific mean values and mean-centered values of valence, calmness and perceived stress were included in multilevel models.

	**Models for disentangling within- and between-person associations between AC and variables of mood and perceived stress Outcome: alcohol consumption**			
Predictor	β coefficient (SE) [95% CI]	t(df)	F-value	p-value
**Valence**				
Intercept	53.30 (6.67)	8.00 (46039)	819.24	<.001
Valence_m	-1.61 (0.63) [-2.85 - -0.37]	-2.55 (356)	6.47	.011
Valence_c	0.57 (0.20) [0.19 - 0.96]	2.90 (46039)	8.43	.004
**Calmness**				
Intercept	48.95 (6.13)	7.99 (46028)	817.21	<.001
Calmness_m	-1.24 (0.60) [-2.41 - -0.06]	-2.05 (356)	4.19	.041
Calmness_c	0.42 (0.17) [0.09 - 0.75]	2.48 (46028)	6.17	.013
**Perceived stress**				
Intercept	35.03 (3.91)	8.95 (35059)	762.72	<.001
PSS_m	0.13 (0.23) [-0.32 - 0.58]	0.55 (326)	0.30	.584
PSS_c	-0.07 (0.12) [-0.30 - 0.17]	-0.55 (35059)	0.30	.583

Equations:

$$Y(AC)_{ij} = \beta_{00} + \beta_{01} * valence\_mean_j + \beta_{10} * valence\_centered_{ij} + \mu_{ij} + r_{ij}$$

$$Y(AC)_{ij} = \beta_{00} + \beta_{01} * calmness\_mean_j + \beta_{10} * calmness\_centered_{ij} + \mu_{ij} + r_{ij}$$

$$Y(AC)_{ij} = \beta_{00} + \beta_{01} * perceivedstress\_mean_j + \beta_{10} * perceivedstress\_centered_{ij} + \mu_{ij} + r_{ij}$$

eTable 23 Results of multilevel models for the AC prediction by PSS scores

The PSS values were mean-centered to control for between-person effects. To control for the influence of other between- and within-subjects variables the original basic model was extended by all covariates. Due to the low temporal resolution of the PSS values (monthly data acquisition), the systematic control for the influence of weekends and holidays has not been performed here.

	**Multilevel models for AC prediction by perceived stress scores**<br>**Outcome: alcohol consumption**			
Predictor	β coefficient (SE) [95% CI]	t(df)	F-value	p-value
**Perceived stress**<br>no covariates				
Intercept	37.60 (2.15)	17.46 (35059)	737.91	<.001
PSS_c	-0.05 (0.11) [-0.27 - 0.17]	-0.46 (35059)	0.21	.649
including all between- and within-subject variables				
Intercept	11.88 (9.81)	1.21 (34033)	813.56	.226
PSS_c	0.00 (0.12) [-0.23 - 0.24]	0.04 (34033)	0.45	.971

Equations:

$$Y(AC)_{ij} = \beta_{00} + \beta_{01} * perceivedstress\_centered_{ij} + \mu_{ij} + r_{ij}$$

$$Y(AC)_{ij} = \beta_{00} + \beta_{01} * age_j + \beta_{02} * gender_j + \beta_{03} * AUDcriteria_j + \beta_{04} * depression_j + \beta_{05} * profession_j + \beta_{06} * graduation_j + \beta_{07} * status_j + \beta_{08} * child_j + \beta_{09} * site_j + \beta_{10} * lockdown_{ij} + \beta_{20} * perceivedstress\_centered_{ij} + \mu_{ij} + r_{ij}$$

eReferences

1. Heinz A, Kiefer F, Smolka MN, et al (2020) Addiction Research Consortium: Losing and regaining control over drug intake (ReCoDe) - From trajectories to mechanisms and interventions. *Addict Biol* 25(2):e12866. doi:10.1111/adb.12866
2. Arend MG, Schäfer T (2019) Statistical power in two-level models: A tutorial based on Monte Carlo simulation. *Psychol Methods* 24(1):1-19. doi:10.1037/met0000195
3. Bolger N, Laurenceau J-P (2013) *Intensive Longitudinal Methods: An Introduction to Diary and Experience Sampling Research*. Guilford Press.
4. Deeken F, Reichert M, Zech H, et al (2022) Patterns of Alcohol Consumption Among Individuals With Alcohol Use Disorder During the COVID-19 Pandemic and Lockdowns in Germany. *JAMA Netw Open*. 5(8):e2224641. doi:10.1001/jamanetworkopen.2022.24641
5. Bundesregierung. Corona: Diese Regelungen gelten ab 2. November. https://www.bundesregierung.de/breg-de/themen/coronavirus/regelungen-ab-2-november-1806818. Updated November 8, 2021.000Z. Accessed November 8, 2021.542Z.
6. Bundesregierung. Lockdown: Diese Regeln gelten ab heute. https://www.bundesregierung.de/breg-de/themen/coronavirus/bundesweiter-lockdown- 1829134. Updated November 8, 2021.000Z. Accessed November 8, 2021.535Z.
7. Bundesgesundheitsministerium. Coronavirus-Pandemie: Was geschah wann? https://www.bundesgesundheitsministerium.de/coronavirus/chronik-coronavirus.html. Accessed July 8, 2022.
8. NDR. Corona-Chronologie: März 2021. https://www.ndr.de/nachrichten/info/Corona-Chronologie-,coronachronologie138.html. Accessed July 8, 2022.
9. NDR. Corona-Chronologie: April 2021. https://www.ndr.de/nachrichten/info/Corona-Chronologie-,coronachronologie140.html. Accessed July 8, 2022.
10. NDR. Corona-Chronologie: Mai 2021. https://www.ndr.de/nachrichten/info/Corona-Chronologie-Mai-2021,coronachronologie142.html. Accessed July 8, 2022.
